# Lipidomic traits of plasma and cerebrospinal fluid in amyotrophic lateral sclerosis correlate with disease progression

**DOI:** 10.1093/braincomms/fcab143

**Published:** 2021-06-26

**Authors:** Joaquim Sol, Mariona Jové, Monica Povedano, William Sproviero, Raul Domínguez, Gerard Piñol-Ripoll, Ricardo Romero-Guevara, Abdul Hye, Ammar Al-Chalabi, Pascual Torres, Pol Andres-Benito, Estela Area-Gómez, Reinald Pamplona, Isidro Ferrer, Victòria Ayala, Manuel Portero-Otín

**Affiliations:** 1 Metabolic Physiopathology Research Group, Experimental Medicine Department, Lleida University-Lleida Biochemical Research Institute (UdL-IRBLleida), Lleida, Spain; 2 Institut Català de la Salut, Atenció Primària, Lleida, Spain; 3 Research Support Unit Lleida, Fundació Institut Universitari per a la recerca a l'Atenció Primària de Salut Jordi Gol i Gurina (IDIAPJGol), Lleida, Spain; 4 Functional Unit of Amyotrophic Lateral Sclerosis (UFELA), Service of Neurology, Bellvitge University Hospital, L'Hospitalet de Llobregat, Barcelona, Spain; 5 Department of Basic and Clinical Neuroscience, King's College London, Maurice Wohl Clinical Neuroscience Institute, London, UK; 6 Cognitive Disorders Unit, Clinical Neuroscience Research, IRBLleida-Hospital Universitari Santa Maria Lleida, Lleida, Spain; 7 Department of Pathology and Experimental Therapeutics, University of Barcelona, Barcelona, Spain; 8 CIBERNED (Network Centre of Biomedical Research of Neurodegenerative Diseases), Institute of Health Carlos III, Ministry of Economy and Competitiveness, Barcelona, Spain; 9 Bellvitge Biomedical Research Institute (IDIBELL), Hospitalet de Llobregat, Barcelona, Spain; 10 Department of Neurology, Columbia University Irving Medical Center, New York, NY, USA; 11 Senior Consultant, Bellvitge University Hospital, Barcelona, Spain; 12 Institute of Neurosciences, University of Barcelona, Barcelona, Spain

**Keywords:** triacylglyceride, hypermetabolism, metabolomics, plasma, CSF

## Abstract

Since amyotrophic lateral sclerosis cases exhibit significant heterogeneity, we aim to investigate the association of lipid composition of plasma and CSF with amyotrophic lateral sclerosis diagnosis, its progression and clinical characteristics. Lipidome analyses would help to stratify patients on a molecular basis. For this reason, we have analysed the lipid composition of paired plasma and CSF samples from amyotrophic lateral sclerosis cases and age-matched non-amyotrophic lateral sclerosis individuals (controls) by comprehensive liquid chromatography coupled to mass spectrometry. The concentrations of neurofilament light chain—an index of neuronal damage—were also quantified in CSF samples and plasma. Amyotrophic lateral sclerosis versus control comparison, in a moderate stringency mode, showed that plasma from cases contains more differential lipids (*n* = 122 for raw *P* < 0.05; *n* = 27 for *P* < 0.01) than CSF (*n* = 17 for raw *P* < 0.05; *n* = 4 for *P* < 0.01), with almost no overlapping differential species, mainly characterized by an increased content of triacylglyceride species in plasma and decreased in CSF. Of note, false discovery rate correction indicated that one of the CSF lipids (monoacylglycerol 18:0) had high statistic robustness (false discovery rate-*P* < 0.01). Plasma lipidomes also varied significantly with the main involvement at onset (bulbar, spinal or respiratory). Notably, faster progression cases showed particular lipidome fingerprints, featured by decreased triacylclycerides and specific phospholipids in plasma, with 11 lipids with false discovery rate-*P* < 0.1 (*n* = 56 lipids in plasma for raw *P* < 0.01). Lipid species associated with progression rate clustered in a relatively low number of metabolic pathways, mainly triacylglyceride metabolism and glycerophospholipid and sphingolipid biosynthesis. A specific triacylglyceride (68:12), correlated with neurofilament content (*r* = 0.8, *P* < 0.008). Thus, the present findings suggest that systemic hypermetabolism—potentially sustained by increased triacylglyceride content—and CNS alterations of specific lipid pathways could be associated as modifiers of disease progression. Furthermore, these results confirm biochemical lipid heterogeneity in amyotrophic lateral sclerosis with different presentations and progression, suggesting the use of specific lipid species as potential disease classifiers.

## Teaser

Sol et al. report that in case–control approaches, plasma lipidomic profile is richer in differences than CSF. Interestingly, patients with faster progression show decreased plasmatic triacylglycerides and phospholipids, compatible with hypermetabolism. They conclude that the plasma lipidomic profile holds promise for patient stratification.

## Graphical Abstract

**Graphical Abstract:**
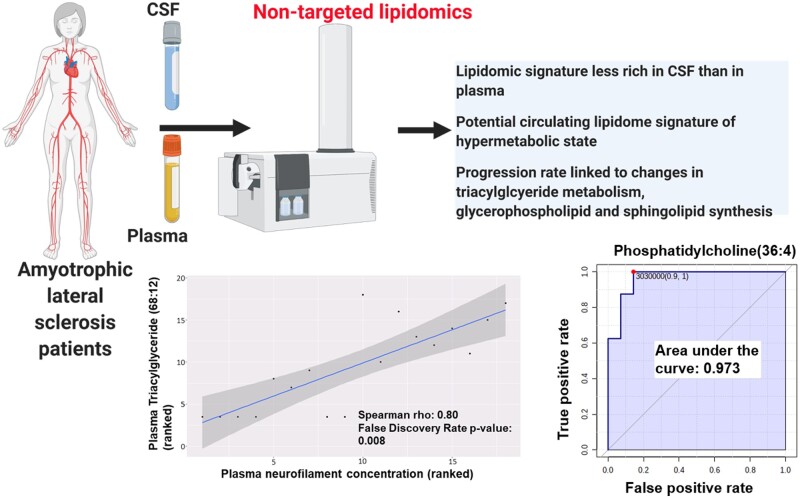

## Introduction

Amyotrophic lateral sclerosis (ALS) is a sporadic or familial, progressive, neurodegenerative disease leading to the gradual loss of motor function, due to the upper and lower motor neurons degeneration and death.^1^ Even considering monogenic forms such as those linked to mutations in *SOD1*, *TDP-43, FUS* and *C9ORF72*, there is high clinical individual heterogeneity, including a variable rate of progression and site of onset.^2^ The causes for the heterogeneity are non-modifiable variables, such as ageing, site of initiation, involved genes and other individual traits,^3^ together with environmental factors, such as smoking, strenuous exercise, exposure to pesticides and metals, and individual care.^4–6^ Indeed, it is well known that metabolic changes in ALS patients—specifically those pointing to hypermetabolism^7^^,^^8^—, broadly defined as increased energy expenditure, are prognosis modifiers.^9^

Markers used for the diagnosis and prediction of disease progression of ALS include different clinical variables,^10^ neuroimaging techniques,^11^ electromyographical studies,^12^ epigenetic alterations,^13^ changes in neurofilament levels in the CSF,^14–16^ serum microRNAs,^17^^,^^18^ C-reactive protein,^19^ urinary p75,^20^ YKL40 levels in the CSF,^20^ and inflammatory blood biomarkers, among other fluid biomarkers (reviewed in Swindell et al.^21^). In addition to these procedures and observations, metabolomics is a powerful tool for a dynamic readout of relevant pathophysiological pathways in different settings.^22^^,^^23^ The subset of metabolomics focussed on the study of lipidome is of particular interest in studying neurodegenerative diseases, such as Alzheimer's disease and age-related neuropathological conditions.^24^

Liquid chromatography coupled to mass spectrometry (LC/MS) may render several hundreds of molecules that could pave the way to discover pathogenic pathways and the identification of new biomarkers. Lipidome analysis of the CSF^25^ and plasma^26^ has recently been reported in ALS cases. However, the parallel study of CSF and plasma from the same individual may provide information about common and differential levels of distinct molecules derived from the CNS or resulting from systemic metabolism.^9^ This interest is based on the recently described traffic of lipids across the blood-to-brain barrier (BBB)^27^ and the potential disturbances of this barrier in ALS.^28^^,^^29^

We assessed plasma and CSF lipidome in the same ALS cases and age-matched healthy individuals in this study. The ALS-associated changes are correlated with relevant clinical data, including the progression rate and levels of other biomarkers, as neurofilament content. Globally, ALS-associated changes in plasma lipidome are more prominent than those in CSF. Furthermore, the results support the use of specific lipids—mainly triacylglycerides (TGs)—to monitor hypermetabolism in relationship with progression rate.

## Material and methods

### Study population and sample collection

The study followed the guidelines of the relevant Spanish legislation (Real Decreto 1716/2011) and enjoyed the Institutional Ethics Committee of the Bellvitge University Hospital's approval. CSF and blood (plasma) were obtained from 23 ALS cases in the Bellvitge University Hospital, Barcelona, between 2015 and 2016, and from 10 age-matched non-ALS individuals who underwent lumbar puncture for CSF extraction as part of the diagnostic procedure at the Santa Maria University Hospital, Lleida, to investigate peripheral polyneuropathy, Guillain–Barre Syndrome or self-resolved neurological symptom (Supplementary Table 1 for clinical characteristics of non-ALS individuals). In all cases, CSF and plasma were obtained in the morning after overnight fasting, according to standardized clinical chemistry laboratory conditions. Before storage, 1 μl of a 100 μM butylhydroxytoluene solution in ethanol was added to 99 μl of sample aliquots as an antioxidant to avoid the artefactual formation of lipid oxidation products. Samples were stored in the first hour after obtention at −80°C. All samples were analysed in the next six months.

Informed consent was obtained from all participants. ALS cases were 13 men (four bulbar, three spinal and six with respiratory onset) and 10 women (three bulbar, one spinal and six with respiratory onsets) (Table 1, Supplementary Table 2 for extended information). Biological samples were obtained between 10 and 24 months after the beginning of symptoms, and ALS was diagnosed according to the El Escorial criteria for ALS. The ALS-functional rate was calculated and validated by two independent neurologists. The progression rate was calculated (at baseline or last visit) as 48 minus the ALS Functional Rating Scale-Revised score, divided by the disease duration from onset of symptoms.^30^ We defined 1.35 (point/month) as the cutoff point for classifying progression rates as fast progressors (FP) or normal progressors (NP).

**Table 1:** Clinical characteristics of ALS cases

Age at diagnosis (years)	60 ± 10
Rate of progression (number of individuals)	
Fast progressors	15
Normal progressors	8
Onset type (number of individuals)	
Bulbar	7
Respiratory	4
Spinal	12
MITOS at diagnosis	
Not determined	5
0	15
1	3
Diagnostic delay (days)	250 ± 189
Plasma cholesterol (mmol/l)	13.41 ± 36.4
Plasma LDL-cholesterol (mmol/l)	2.92 ± 1.05
Plasma HDL-cholesterol (mmol/l)	1.02 ± 0.61
Plasma tryglicerides (mmol/l)	6.94 ± 25.44
CSF-Glucose (mmol/l)	3.52 ± 0.51
CSF-Protein concentration (g/l)	0.37 ± 0.10

MITOS, Milano-Torino functional staging. Continous data are expressed as mean values ± standard deviation.

### Chemicals

Synthetic lipids were from Avanti Polar Lipids Inc. (Alabaster, AL) and Sigma–Aldrich (Madrid, Spain). We employed the following LC/MS quality-grade solvents: methyl tert-butyl ether, acetonitrile, isopropanol, potassium chloride, chloroform, ammonium formate and ammonium hydroxide, all purchased from Sigma–Aldrich (Madrid, Spain); methanol was from Carlo Erba (Milano, Italy); acetone was from Riedel-de-Häen (Seelze, Germany); and formic acid was from Baker (Phillipsburg, NJ, USA).

### Lipidomic analysis

#### Preparation of lipid standards

Lipid standards consisting of isotopically labelled lipids were used for external standardization (i.e. lipid family assignment) and internal standardization (i.e. for adjustment of potential inter-and intra-assay variances). Stock solutions were prepared by dissolving standards in methyl tert-butyl ether (MTBE) at a concentration of 1 mg/ml, and working solutions were diluted to 2.5 µg/ml in MTBE (Supplementary Table 3). These internal standards of each lipid class are added before lipid extraction (see below).

#### Lipid extraction

The lipidomic analysis was based on a previously validated methodology.^31^ Five microlitres of Mili Q water and 20 µl of methanol were added to 10 µl of the sample (either plasma or CSF) and vortex-mixed for 2 min to precipitate proteins. For lipid extraction, 250 µl of MTBE containing internal standards was added, and tubes containing the samples were immersed in a water bath (ATU Ultrasonidos, Valencia, Spain) with an ultrasound frequency and power of 40 kHz and 100 W, at 10°C for 30 min. Then, 75 µl of Mili Q water was added to the mixture, and the organic phase was separated by centrifugation at 3000× rpm at 10°C for 10 min. 170 µl of the upper phase-containing lipid extracts were collected and stored in vials at −20°C. A pool of 30 µl obtained from all samples was used as quality control (QC sample) in multivariate analyses.

#### LC–MS method

Lipid extracts were analysed by LC/MS using a liquid chromatograph Agilent UPLC 1290 coupled to a mass spectrometer Agilent Q-TOF MS/MS 6520 (Agilent Technologies, Barcelona, Spain). The analysis was based on a published method.^31^ The order for the injection of samples was randomized, and QC samples were distributed every seven samples to control instrumental drift. The sample compartment was refrigerated at 4°C. For each sample, 10 µl of lipid extract was injected in the system onto a 1.8 µm particle 100 × 2.1 mm id Waters Acquity HSS T3 column (Waters, Milford, MA, USA), and heated at 55°C. Gradient elution consisted of two phases; phase A was composed of 10 mM ammonium acetate in acetonitrile–water (40:60, v/v), and phase B of 10 mM ammonium acetate in acetonitrile–isopropanol (10:90, v/v). These were introduced to the system at a constant flow rate of 400 µl/min. The gradient started at 40% B and reached 100% B in 10 min, and was maintained for 2 min. Finally, the system was switched back to 60% B and then equilibrated for 3 min. Data from electrospray positive and negative ionized species were obtained in duplicate runs of the samples. The system operated in TOF full-scan mode, at 100 *m/z* to 3000 *m/z* in an extended dynamic range (2 GHz), using N_2_ as nebulizer gas (5 l/min, 350°C). The capillary voltage was set at 3500 V with a scan rate of 1 scan/s. Continuous infusion using a double spray with masses 121.050873, 922.009798 (positive ion mode), and 119.036320, 966.000725 (negative ion mode) was used in-run continuous calibration of the mass spectrometer. For MS/MS confirmation, the same parameters used for MS analyses were utilized, adding collision voltages of 0 V, 10 V, 20 V and 40 V. Data were collected with MassHunter Data Acquisition software (Agilent Technologies, Barcelona, Spain). Relative SD was calculated from the intensity of these internal standards across the QC following,^32^ and median values of this relative SD allowed us to estimate the interassay and intraassay coefficient of variances, which was below 9%.

### Neurofilament analyses

CSF and plasma neurofilament (NfL) concentration was measured using the Simoa platform (NF-light; Quanterix, Lexington, MA, USA) at the Maurice Wohl Clinical Neuroscience Institute (London, UK). Samples were randomized, blinded and measured in duplicate using a batch of reagents from the same lot. The intra-assay error was 8.1%. The detection limit was 0.52 pg/ml and the lower limit of quantification 3.26 pg/ml, compensated for 4-fold sample dilution. Sample concentrations were calculated on the Simoa software and exported for further statistical analysis.

### Data processing and statistic analyses

Before data processing, we checked each lipid standard's relative abundances in all the samples for the quality assessment using specific software. All samples shown passed this quality assessment, supporting the robustness of the analyses. According to the specific metabolomics software, the intensity of features (measured as arbitrary units) is normalized using one representative internal standard (MassProfiler Professional, Agilent). The sample with the highest internal standard abundance is used as a reference, and the other samples are scaled, multiplying by the relative internal standard abundance of each sample compared to the reference. All investigation was performed blinded: we performed statistical analyses, not knowing specific clinical characteristics. Thus, statistical analyses were blinded with respect to the identity of the groups being compared. After all the analyses were completed, identities of clinical characteristics (i.e. non-ALS versus ALS, onset and progression rate) were attributed to the corresponding samples after grouping the data. Molecular features (i.e. groups of ions with similar chromatographic behaviour and compatible with the same molecular composition, accounting adduct formation) were extracted with MassHunter Qualitative Analysis (Agilent Technologies, Barcelona, Spain), as previously detailed.^33^ Molecular features in the same retention time [i.e. within a 0.1% of total run time (±0.25 min)] and in the same mass window [i.e. within 30 ppm (±2 mDa)] were considered the same to account for instrumental drift. We chose the 30 ppm tolerance based on actual conditions obtained by authentic standards. Only common features (found in at least 50% of the samples of the same condition compared) were considered to minimize individual bias. Peak intensities were relativized by internal standard peak intensity. In order to choose the most reliable identity of the lipid when there was no MS/MS confirmation and there was more than one option, we followed the following criteria according to previous experience and the composition of the mobile phases: for positive ionization, M+H, M+NH_4_, M+H−H_2_O, and M+Na were employed; for negative ionization, M−H, and M+CH_3_OO were used. Mass to charge ratios (*m/z*) shown are those majoritarian in the ion profile of given species, and they could be derived from molecular ion (i.e. +H^+^ in positive ionization or –H+ in negative ionization) or ionization of adducts (e.g. +NH_4_^+^ in positive ionization or +COOH^−^ in negative ionization).

Text files (.txt) containing relative intensities of every metabolite in every sample were formatted for later statistical analysis with specific R-based scripts. Non-parametric tests (Mann–Whitney and Kruskal–Wallis tests), Spearman correlations and multivariate analyses [principal component analyses (PCA) and hierarchical clustering] were performed with these scripts, and ROC curves were obtained using Metaboanalyst software.^34^ In univariate analyses, Benjamini–Hochberg correction was adopted to correct for type 1 error, being shown as false discovery rate (FDR) corrected *P*-value. Based on the semiquantitative nature of non-targeted lipidomics, fold-change differences are rounded to <0.1 or >10 in the figures to avoid the introduction of overestimation of differences. Exact fold-changes are presented in the Supplementary Data set 1.

### Annotation and pathway analysis

Molecules showing statistically significant expression (with *P*-value < 0.05 in differential analyses) were annotated by comparing their exact mass and retention time and isotopic distribution with specific databases^35^^,^^36^ to obtain potential identities. Identities were confirmed by comparing resulting MS/MS spectra against representative class standards using the LipidMatch workflow.^37^ The MS/MS spectrum allowed the annotation of lipid families (e.g. glycerophospholipids) based on the presence of specific fragments. For example, the appearance of *m/z* = 184 was interpreted as its precursor being a glycerophosphatidylcholine or sphingomyelin, while neutral losses of 141 indicated glycerophosphatidylethanolamine. Please note that in most cases, this method precludes the identification of the exact acyl chain composition. Annotation of lipid species has been performed based on exact mass and retention time, which allows the acyl chain's total sum report. The proposed acyl chain composition is based on MS/MS spectra analyses when specified in the results. In all cases, results and discussion comprise both positively and negatively ionized lipids.

Pathway analyses were performed using the LIPEA platform^38^ and the *Homo sapiens* as lipidome background, and by employing the enrichment analysis option in the Consensus PathDB-human platform^39^ against the Kyoto Encyclopedia of Genes and Genomes database,^40^ with a minimum overlap of two lipids with those of reference, and a *P*-value cutoff of 0.01.

### Data availability

Data employed for making figures are available as Supplementary datasets. Raw data are available from the authors on request.

## Results

### Plasma lipidome, in comparison with CSF, defines a more evident ALS signature

**Figure 1:**
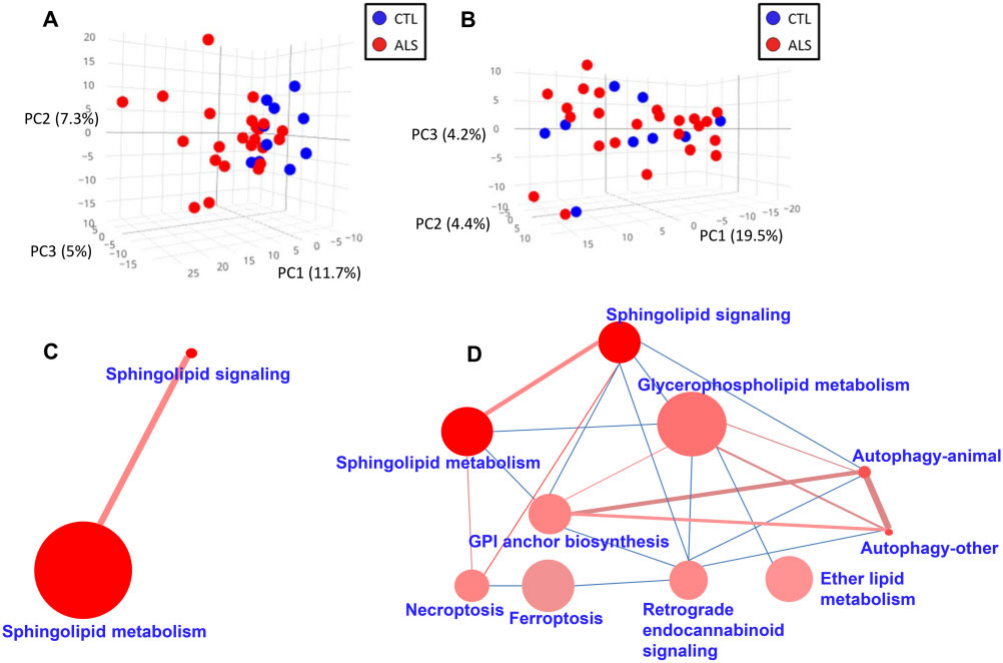
Lipidomic profiles in plasma and CSF in ALS patients in comparison with non-ALS individuals. Lipids from plasma (**A**) and CSF (**B**) define a graph of principal component analysis (PCA). Sample grouping is displayed with different colours: CTL in blue and ALS in red. These results show molecular findings detected in positive ionization. Lipid-enriched pathways are shown by mapping differentially down-regulated (**C**) and upregulated (**D**) lipids in plasma in ALS. The size of the nodes (pathways) is proportional to the total number of metabolites belonging to the pathway; the colour intensity is inversely proportional to the *P*-value of the enrichment analyses; the edge width, directly proportional to the number of common metabolites, and the edge colour intensity, directly proportional to the number of lipids differentially regulated in ALS (*N* = 23) versus CTL (*N* = 10) cases. These results represent lipids detected by positive ionization.

**Figure 2:**
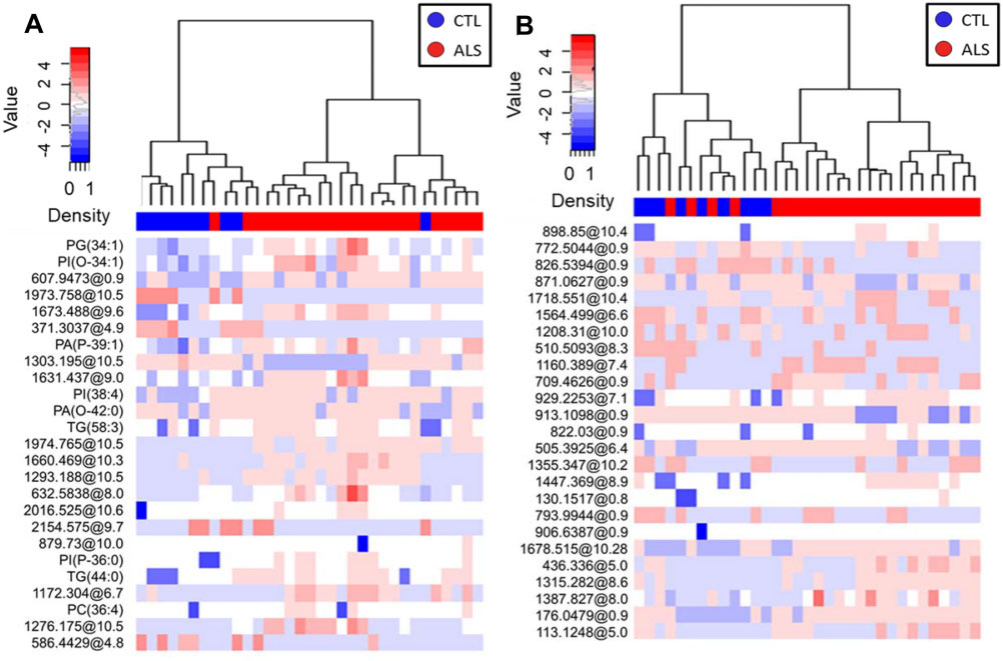
Lipidomic profiles in plasma and CSF allow clustering of ALS patients and non-ALS individuals. Hierarchical clustering using the 25 lipids with the lowest *P*-value obtained from univariate statistics comparing plasma (**A**) and CSF (**B**) from ALS versus CTL. Each line of the heatmap represents a lipid species coloured by its abundance intensity normalized with an internal standard, log-transformed, and row-normalized using *Z*-scores. The scale from blue to red shows the normalized abundances in MS counts. Samples are organized in columns and ordered according to the hierarchical clustering results. We employed the Ward clustering method and Manhattan distance for hierarchical clustering. Patient groups in dendrograms are displayed with different colours: CTL (*N* = 10) in blue and ALS (*N* = 23) in red. These results show molecular findings detected in positive ionization.

The lipidome analyses of plasma and CSF samples revealed 1018 specific lipid species in plasma, 843 in CSF. Multivariate and univariate statistics were performed (Fig. 1, Supplementary Table 4). Our results showed that the effect of ALS in lipid profile was much more marked in plasma than in CSF, as demonstrated by PCA (Fig. 1A and B, Supplementary Fig. 1 for negatively ionized lipids). In plasma, with a low stringency statistics, 122 differential lipids (raw *P* < 0.05, Mann–Whitney test; 27 with raw *P* < 0.01) were found comparing ALS cases and controls (66% of them increased in ALS) (Supplementary Data set 1), 53 of them (44%) annotated (Supplementary Table 4). These lipids belonged mainly to the glycerolipid (21/53, 40%) and glycerophospholipid (25/53, 47%) families. In contrast, in CSF, only 17 lipids differed between ALS cases and controls (raw *P* < 0.05, Mann–Whitney test, 4 with raw *P* < 0.01), one of them with FDR *P*-value < 0.1, 24% upregulated in ALS (Supplementary Dataset 1), with five of them identified (Supplementary Table 4). The hierarchical clustering approach using only 25 lipid species with the lowest *P*-values (Fig. 2A and B) reinforced the lack of discriminatory power in CSF samples, where four CSF ALS samples aggregated better with the control than with the ALS group. A sunburst type interactive graph showing these differential lipids is available online (http://alslipidomedgn.pythonanywhere.com/).

Differentially down- and upregulated clusters in ALS plasma are shown in Fig. 1C and D, respectively. Globally, these differential lipids are clustered among several pathways, including *Sphingolipid metabolism, Autophagy, Necroptosis, Choline metabolism in cancer,*

*Sphingolipidsignalling, Sphingolipid metabolismand Neurotrophinsignallingpathway* (Supplementary Dataset 2). Notably, no overlapping differential lipid profiles were observed when comparing CSF and plasma.

### ALS region onset identifies different plasma and CSF lipidomic profiles

Since disease onset is a clinically relevant variable, we examined whether cases differing in the onset site had specific lipidomic profiles. PCA analyses showed no clustering of plasma and CSF samples when considering disease onset (Supplementary Figs 2 and 3), with higher homogeneity in the bulbar group (Supplementary Fig. 2A). However, a precise hierarchical clustering was defined using the 25 plasma lipid species with the lowest *P*-value obtained from univariate statistics (Supplementary Fig. 2C). The two main clusters comprised the group with the spinal onset and the groups with bulbar plus respiratory onset, potentially defining different metabolic patterns. In contrast, in CSF, only the spinal group was well defined (Supplementary Fig. 2D).

Univariate statistics with moderate stringency revealed 38 significantly different lipids in plasma (raw *P* < 0.05, Kruskal–Wallis test, 8 with raw *P* < 0.01) between the three groups of ALS cases regarding the site of disease onset (14 of them were identified), and 17 (1 with *P* < 0.01) in the CSF (5 identified), with glycerolipids and glycerophospholipids being the most abundant families (Supplementary Dataset 1, Supplementary Table 5). Of note, FDR correction did not include these lipids.

Also, to evaluate whether these changes were related to locomotor activity or food intake, we analysed plasma creatine kinase and body-mass index (BMI). Significant differences were encountered between the three groups (Supplementary Table 6), with spinal site onset cases showing lower than the usual BMI and those with respiratory onset lower creatine kinase concentrations.

### Lipidomic profiles differentiate ALS progression rate

**Figure 3:**
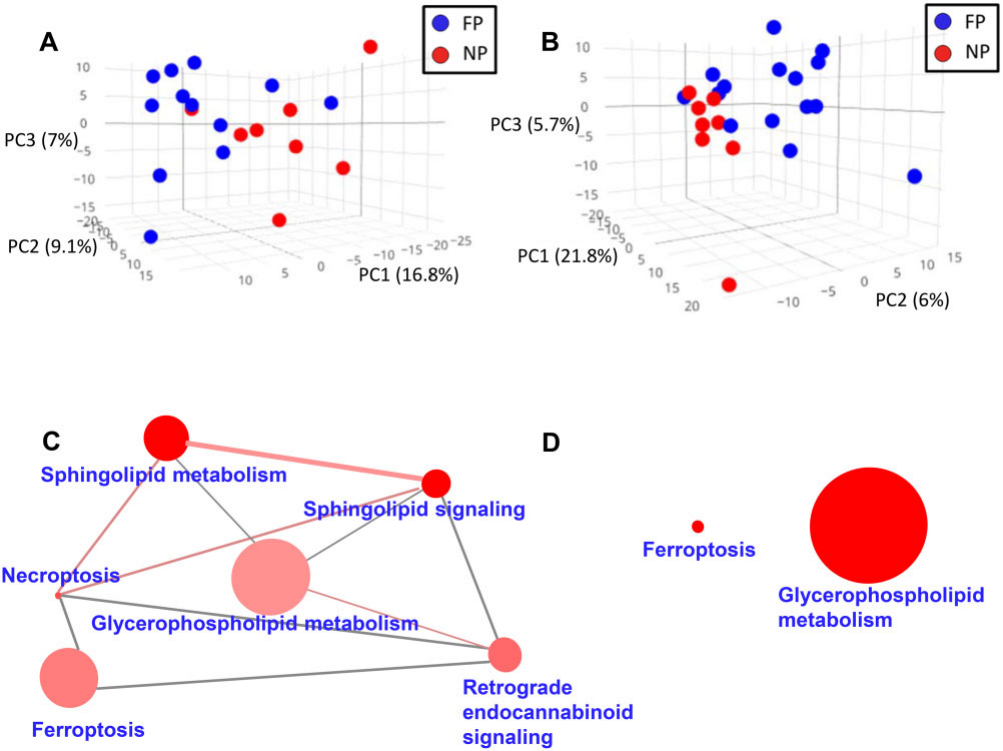
Lipidomic profiles in plasma and CSF and disease progression in ALS. Plasma (**A**) and CSF (**B**) lipidomic profiles in fast progressor (FP, in blue) versus normal progressors (NP, in red). Lipids from each fraction define a graph of PCA with marked differences in plasma and CSF. Results represent molecular findings detected in positive ionization. Lipid-enriched pathways differentially down-regulated (**C**) and upregulated (**D**) lipids in plasma. The size of the nodes (pathways) is proportional to the total number of metabolites belonging to the pathway; the colour intensity is inversely proportional to the p-value of the enrichment analyses; the edge width is directly proportional to the number of common metabolites, and the edge colour intensity, directly proportional to the number of lipids differentially regulated in FP (*N* = 15) versus NP (*N* = 8). These results represent lipids detected by positive ionization.

**Figure 4:**
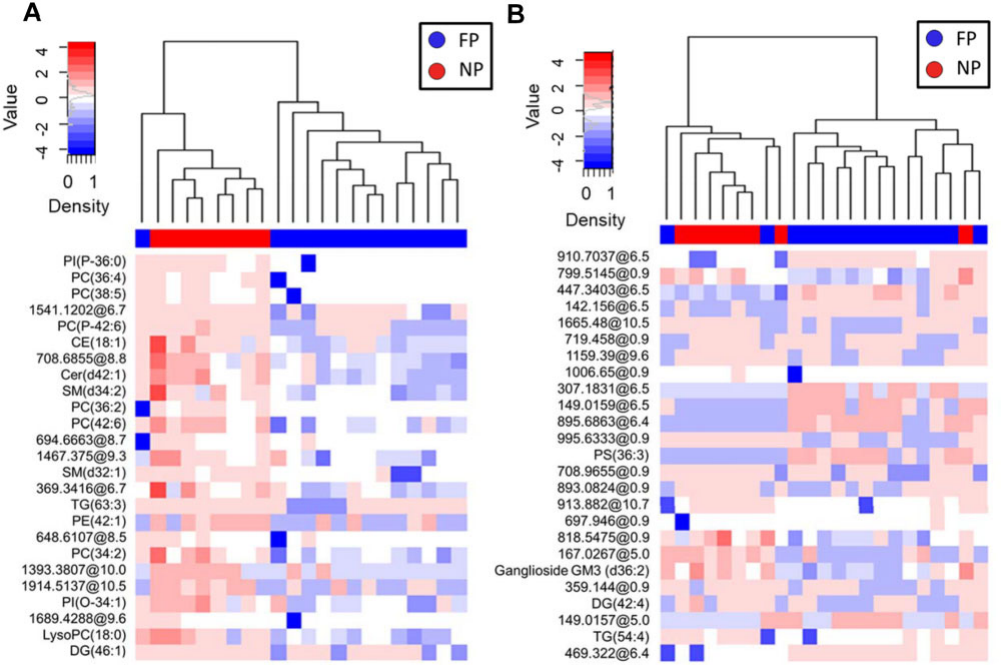
Lipidomic profiles in plasma and CSF allow clustering according disease progression in ALS. Plasma (**A**) and CSF (**B**) lipidomic profiles in fast progressor (FP) versus normal progressors (NP) were analized employing hierarchical clustering using the 25 lipids with the lowest *P*-value obtained from univariate statistics comparing FP versus NP. Each segment of the graph in heatmaps represents a lipid species coloured by its abundance intensity, normalized to an internal standard, log-transformed, and row-normalized using *Z*-score. The scale from blue to red represents these normalized abundances in MS counts. We employed the Ward clustering method and Euclidean distance for hierarchical clustering. Samples are organized in columns and ordered according to hierarchical clustering. Dendrograms and sample grouping are displayed in different colours: FP (*N* = 15) in blue and NP (*N* = 8) in red. Results represent molecular findings detected in positive ionization.

As hypermetabolism could lead to changes in plasma lipids and be linked to faster progression rates,^41^ we evaluated the potential differences in ALS lipidomic profiles according to progression rate, as defined in the Material and Methods section. The lipidomic pattern of plasma and CSF showed different profiles depending on the ALS progression rate (Fig. 3, Supplementary Fig. 4 for negatively ionized lipids). Again, differences in plasma were more marked than those in CSF (Figs 3A–B and 4).

As shown in Supplementary Dataset 1, levels of 190 lipids were significantly different between FP and NP in plasma (raw *P* < 0.05, Mann–Whitney test, 56 with raw *P* < 0.01), 11 of them with FDR *P*-value < 0.1. Among the 106 lipid species with a potential identity (based on exact mass, retention time, isotopic distribution and M/MS spectrum), 48 were glycerolipids (involving 40 triglycerides) and 45 glycerophospholipids. Of note, 85% of the differential molecules were decreased in the FP group, suggesting the progression rate is linked to the depletion of specific lipids (Supplementary Table 7). In the CSF, the levels of 63 lipids were significantly different between the FP and NP groups (raw *P* < 0.05, Mann–Whitney test, 9 with raw *P* < 0.01); 68% of them were decreased in FP (Supplementary Dataset 1). In this case, FDR did not disclose any differential CSF lipid. Differential lipids in plasma clustered among several metabolic pathways, such as *ferroptosis, glycerophospholipid metabolism, sphingolipid metabolism, necroptosis and sphingolipid signalling pathway* (Fig. 3C and D; Supplementary Dataset 2; Supplementary Table 7 and Supplementary Fig. 4). A sunburst type interactive graph showing these differential lipids is available online (http://alslipidomeprog.pythonanywhere.com/). No significant differences were found in BMI or the levels of plasmatic creatinine kinase between NP and FP cases (Supplementary Table 8), suggesting that these changes were independent of the locomotor activity or food intake.

We should note the occurrence of odd-numbered carbon acyl content lipids, among the differential lipids found in plasma (and in CSF to a lower extent). Thus, according to the case–control approach, across the differential lipids in plasma, we found potentially 2 glycerolipids, and 1 glycerophospholipid with odd-numbered carbon acyl content. The potential presence of odd-chain acyl is also evidenced in those differential lipids according to the progression rate. Thus, we found potentially 7 differential glycerolipids in plasma and 1 in CSF, with 1 glycerophospholipid in CSF containing odd-numbered acyl chains. However, please note that MS/MS analyses would be required to confirm their identity as odd-chain acyl lipids.

The relatively high prevalence of lysophospholipids among the detected differential molecules may be related to the very high ionization capacity of these lipids compared with other lipid species. Since the internal standards of glycerophosphatidyl species did not show any significant degradation during extraction procedures (i.e. the occurrence of derived lysophospholipids), we assume that unwanted hydrolysis (e.g. remaining lipase activities) are not contributing significantly to the reported results.

### Lipids defining clinical forms are specific

We evaluated whether differential lipids were the same across the comparisons presented to identify specific lipids associated with specific clinical forms. Only eight differential lipids in plasma in diagnosis (i.e. ALS versus control comparison) overlapped the three affected regions at onset: spinal, bulbar and respiratory in plasma, with 4 of them being annotated (Supplementary Fig. 5A). Thus, respiratory ALS was characterized by increased phosphatidic acid with an ether bound fatty acid [Phosphatidic acid (O-42:0) and increased diacylglycerol (34:1)], whereas bulbar ALS showed characteristically increased triacylglycerol content [TG (58:11)].

Similarly, only eight differential lipids overlapped in the prognostic approach (i.e. FP versus NP comparison) and the three affected regions at onset; five were found in plasma and three in the CSF (Supplementary Fig. 5B). The lipids common to the prognostic approach belonged to the phosphatidylglycerol, phosphatidic acid and TG families. Globally, these comparisons suggest the existence of a lipidomic signature, helping to define each of the clinical subgroups present.

**Figure 5:**
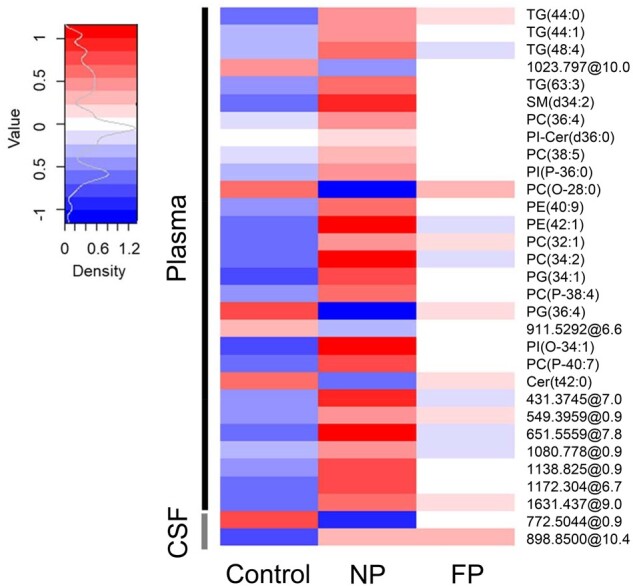
Regulation of common lipid species in ALS diagnosis and disease progression. Heatmap of concentration of common lipids which are differential in the ALS versus non-ALS and disease progression (FP versus NP). Each segment of the heatmap represents the mean value of the lipid species abundance coloured by its abundance intensity, normalized to an internal standard, log-transformed, and row-normalized using *Z*-score. The scale from blue to red represents these normalized abundances in MS counts. Groups of patients are organized in columns.

**Figure 6:**
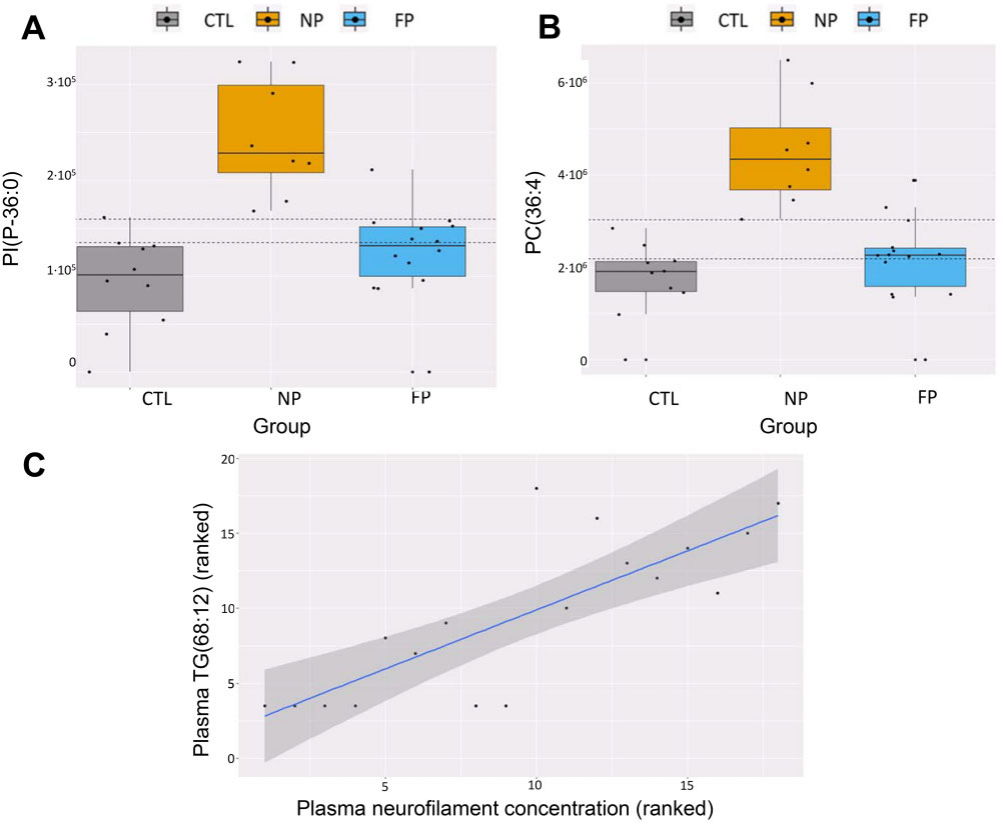
Oppositive regulation of lipid species in ALS diagnosis, disease progression, and relationship with NfL concentrations. (**A**) and (**B**) boxplot showing the levels of the two lipid species with the lowest *P*-value, PC(44:8) and PC(36:4). CTL is represented in grey, NP in orange and FP in blue. Dashed horizontal lines represent optimal cutoff points for classification. (**C**) Relationship between the polyunsaturated TG(68:12) present in plasma samples from ALS patients with the levels of neurofilaments in plasma (*N* = 23). Spearman correlation results are represented as a linear model on the ranked data.

When disease progression biomarkers were compared with ALS diagnosis, we found that 31 of them were common between comparisons (Fig. 5). Interestingly, there is an inverse regulation in ALS diagnosis and disease progression in all of these lipid species, mainly belonging to glycerophospholipids (1 PA, 5 PC, 4 PE, 3 PG, 1 PI, 2 PS) and glycerolipids (5 TGs) families. Molecules with the lowest *P*-value (FDR < 0.1) comprise two phosphatidylcholines [PC(44:8) and PC(36:4)] with high discriminative capacity. Classification accuracy of 72% was obtained for both biomarkers when selecting optimal cutoff points for the classification between control, NP, and FP, in which lower PC levels were associated with the control group and higher levels were associated with NP (Fig. 6A and B). Furthermore, ROC analyses focussed only on the comparison between NP and FP showed that these two lipids are potential biomarkers of the velocity of disease's progression, with areas under the curve (AUC) higher than 0.97 (Supplementary Fig. 6), though we should remind that this has been performed in the same sample cohort were these lipids were discovered. Thus, overfitting of the model could be a drawback of the ROC approach in this context.

### Neurofilament levels correlate with specific lipid species

Neurofilament concentrations in plasma and CSF are associated with ALS progression. We evaluated if there were specific lipids correlated with neurofilament levels. Results showed 98 plasma lipid species significantly correlated (77 negative and 21 positive correlation) with plasma NfL (*P*-value: 6.5E-5–0.05, Spearman coefficient correlation: 0.45–0.77). Among them, only the highly polyunsaturated TG (68:12) presents an FDR *P*-value < 0.1 (Fig. 6C), showing a positive correlation, with a correlation coefficient of 0.80. Concerning CSF, 30 lipid species presented significant correlations with NfL (*P*-value: 0.02–0.05, Spearman coefficient correlation: 0.5–0.72), 26 of them showing a negative and four a positive correlation. In CSF, no correlations had statistically significant FDR values.

## Discussion

One of the most salient features in this work is that plasma lipidomes are quantitatively more affected than CSF lipidomes, which is an unexpected finding considering that ALS is mainly manifested as a neurodegenerative disease. This finding seems not explained by a lower variety of lipids in CSF. Instead, these results point to a general process ongoing with ALS pathogenesis and, particularly, with the development of a hypermetabolic state. This state is defined, in ALS patients, as an unbalance in energy homeostasis,^42^ with calorie uptake often lowered,^43^ and energy expenditure increased in a proportion of patients.^44^ This situation often leads to reduced fat depots,^45^ and this alteration may be associated with increased TGs in plasma. Although plasma lipid levels can be modified by changes in diet and physical activity, from the comparison of progression rates, we found no shreds of evidence of differential BMI or creatine kinase concentration across different groups of patients evaluated here. This lack of global influences supports the fact that the plasma lipidome differences are not merely related to physical activity changes, dietary intake, or similar factors. Thus, plasma lipidome in ALS can be a window for monitoring ALS-associated metabolic changes, thereby helping patients' stratification. Although most of the changes exhibit a high risk for type I error, FDR correction does not preclude that lipids associated with progression are preferentially present in plasma. However, FDR correction in case versus control approach highlights a CSF lipid, the monoacylglycerol (18:0), with diminished values in samples from ALS patients. This decrease would support an enhanced consumption of this lipid, perhaps reflecting increased *de novo* TG synthesis, or it may reveal decreased production by lipases, or diminished transport from blood. Indeed, recent data show that lipid metabolism in the nervous system is compromised, with enhanced glycerophospholipid synthesis to allow axon growth in axon injury situations at the expense of decreased glycerolipid production.^46^ Indeed, in this case, a monoacylglycerol specie—in this case a monoacylglycerol (18:1)—was found to be among the differential lipids downregulated by *lip1* silencing in neurons. In contrast with these data suggesting increased TG synthesis, lipoprotein lipase is upregulated in the symptomatic stage of the G93A mice model of motor neuron disease.^47^ All in all, this would suggest that the above indicated rewiring mechanisms could operate, trying to redirect lipid synthesis towards membranal (i.e. phospholipid) formation.

The specificity of CSF changes and plasma variations is further supported by the almost total absence of common differentially annotated lipids between the two compartments. The lipid content (10–13 µg per ml) of human CSF is *ca* 500 times lower than that of plasma.^48^ The number of lipids detected in CSF in the present study is in the same order of magnitude of independent, comprehensive LC/MS CSF analyses.^49^ This difference in concentration would have led to the leakage of species in plasma to CSF, even in a minimum number of species, more when accounting lipid solubility as a significant factor in determining BBB permeability, and the existence of potential BBB losses of integrity linked to ALS.^28^^,^^29^ The absence of common differential lipids in both fractions reinforces that lipid composition in CSF is a homeostatic variable under tight regulation, even in ALS. Half a litre of CSF is produced daily in the choroid plexus,^50^ ensuring constant lipid supply for neurotransmitter building blocks and membrane and myelin turnover.^51^

Regarding the influence of specific plasma lipids on CSF lipid composition, only recently *in vivo* data have been available in humans. Thus, an infusion of TG in plasma only increased diacylglycerol concentration in CSF humans, without changes in the infused species.^52^ These data support the active role of choroid plexus and other BBB components in maintaining a given lipid composition in CSF.

The present work's findings revealed that the most abundant lipids (glycerolipids and glycerophospholipids) are those contributing to the greatest extent of ALS differentiation. Regarding glycerolipids, ALS cases have increased levels of specific TG species in plasma to cover needs after hypermetabolic states via increased *de novo* hepatic biosynthesis and delivery from adipose tissue depots to skeletal muscle. Of note, in ALS experimental models, there is a metabolic switch towards increased skeletal muscle consumption of lipids preceding motor neuron death.^53^ In line with these results, TG is the lipid family showing the highest number of changes in the spinal cord of ALS G93A transgenic mice.^54^ Furthermore, TG(68:12) concentration correlates with neurofilament levels—a well-known neuron/axon damage marker- in plasma. In comparison with HDL, VLDL and LDL are enriched in TGs. Furthermore, LDL/HDL ratios are increased in this disease, as shown previously.^55^^,^^56^ Our results, showing decreased sphingolipid concentrations in ALS, would agree with decreased HDL concentration, one of the primary plasma sphingolipid vehicles. This specific profile (increased TGs) could sustain hypermetabolism of skeletal muscle.^42^^,^^53^

Many other abundant lipid family members, the glycerophospholipids—major membrane components—are differential between ALS and non-ALS. Our observations agree with previously reported gene expression analyses in the spinal cord, in which several processes related to the homeostasis of membrane lipids were significantly altered in samples from ALS cases.^57^ These pathways included *regulation of lipid kinase activity*, *integral component of luminal side of endoplasmic reticulum membrane*, *endolysosome membrane* and *clathrin-coated endocytic vesicle membrane*. Furthermore, glycerophospholipids are the lipid family most affected by disease progression and contribute to ALS presentation differences, in common with other analyses previously performed in ALS cases.^25^ TG and phospholipid metabolism are tightly cross-regulated,^58^^,^^59^ so it is not surprising that both types of lipids could show changes induced by common metabolic disarrangements.

Concerning ALS progression, our findings showed different lipid profiles in ALS cases with FP when compared with normal progressors. Again, several glycerophospholipids and glycerolipids, as well as sphingolipids (ceramides and sphingomyelins) and cholesteryl esters (CE), are the main contributors to these differences. Interestingly, most of the lipids contributing to differences in the disease's progression rate are lower in fast-progressing individuals, in plasma, and CSF, suggesting increased consumption of these lipids, particularly glycerolipids as TG, primary bioenergetic sources. Furthermore, the fact that plasma TGs are mainly decreased in FP versus NP individuals suggest: (i) that ALS increased TG levels in plasma is a compensatory response, and (ii) the loss of this response could be harmful. In contrast, in case–control comparisons, TG levels are decreased in the CSF of ALS cases, which may be consistent with increased consumption by glial cells and neurons. Of note, decreased TG levels are also present in CSF from FP cases. We described 16 different species that were differentially regulated between ALS diagnosis comparison and ALS progression concerning glycerophospholipids. Among them, two PC presented FDR values< 0.05: PC(44:8) and PC(36:4) and arose as a suitable biomarker candidate to discriminate FP and NP (AUC > 0.97). In line with our results, increased PC(36:4) levels in CSF have been previously described as the most relevant lipid species to discriminate ALS cases.^25^

The occurrence of few odd-numbered carbon acyl lipids across the differential lipids might agree with previous data.^25^ Experimentally, odd-chain lipids exhibit a consistent anaplerotic potential in the brain,^60^^,^^61^ avoiding off-target effects of beta-oxidation. Thus, preferential consumption of neuronal tissues via alpha oxidation might explain these otherwise minority lipids' diminished occurrence across the differential species detected in CSF. Interestingly, silencing *lip1,* a gene related to lipid responses to axonal damage also involved changes in the concentration of several odd-numbered fats, such as PC and PE^46^ at a neuronal level. Nonetheless, we should remind that it is generally considered that odd-chain numbered fatty acids present in animal tissues are obtained from dietary sources.^62^

We have not found significant alterations in the levels of cholesterol or related molecules in ALS, but cholesteryl ester molecules [CE(18:2), CE (18:1), and CE(22:5)] are significant markers of disease progression. Findings in a transgenic model^54^ suggest a pathogenic lipid droplet role (composed of TGs and CE). Interestingly, the concentration of CE(22:5) was decreased in the plasma of FP individuals. This fact agrees with alterations in n-3 metabolism previously suggested in the pathogenesis of the disease,^63–65^ a fact further reinforced by the loss of highly unsaturated TG (56:12; 65:12, 60:9, 68:12) in the plasma of FP cases. Decreased concentration of many TGs containing a high amount of unsaturations is compatible with decreased *de novo* synthesis in these FP cases, as this anabolic process employs preferentially 16:1 and 18:1 as substrates. Furthermore, these may arise from decreased mobilization from adipose tissue depots. As discussed above, the elevations of TGs may be protective in ALS.^66^

Again, these results agree with previous reports showing faster rates of functional decline and lower survival rates in ALS with hypermetabolism,^67^ suggesting that decreased concentrations of specific TGs could be markers of hypermetabolism in humans. The relationship of hypermetabolism with faster progressions has been confirmed in ALS experimental models, such as *SOD1G93A* transgenic mice, where glycolysis, β-oxidation, and mitochondrial metabolism are altered in the spinal cord before disease onset.^68^ Our data show that glycerolipid metabolism could play an essential role in the relationship between hypermetabolism, disease progression, and neurodegeneration.^69^^,^^70^

Furthermore, highlighting the relevance of lipidome analyses, many other lipid families show differential responses across the cases. As an example, our data show lipids potentially annotated as ganglioside GA2 and ganglioside GM3 define CSF lipidomic profiles differentiating ALS clinical forms and disease progression. Ganglioside metabolism has been involved in the pathogenesis of ALS and in other neurodegenerative diseases affecting neurons with longer axons, such as hereditary spastic paraplegia and hereditary sensory neuropathy type 1.^71^

Globally, present observations underscore the importance of lipid metabolism in ALS pathophysiology. Specifically, the present work demonstrates that plasma lipidome can better discriminate ALS diagnosis and progression than CSF. Further, it highlights glycerolipids and glycerophospholipids as the most critical lipid families involved in this pathogenesis. Interestingly, the metabolic adaptation (hypermetabolism) observed in ALS cases is lost in FP, suggesting that this compensatory response's pitfalls are noxious.

As for limitations of our work, we acknowledge the relatively low number of individuals explored, the lack of a replication population, and the potential confounding effects of differential medications, physical activities and diet—to name a few—between ALS and non-ALS individuals (e.g. cases with bulbar origins may have different food intake in terms of both quantity and quality). We should note also that non-ALS individuals underwent lumbar puncture for several neurological symptoms. Thus, we cannot exclude that in the ALS versus non-ALS comparison some of the heterogeneity reported here could be derived from potential influences of the underlying neurological conditions in the non-ALS population. Therefore, even that we offer the FDR values for the comparative analyses, we should remind that this small size may lead to a high rate of type 1 error, and a part of the results might be stochastical. Nonetheless, individuals with fast progression may exhibit profound differences in metabolism that could contribute to changes in lipidome. Both sarcopenia and fat loss would explain, at least in part, differences between individuals with fast progression. However, the levels of a muscle lysis biomarker, creatine kinase, were not differential between FP and NP. Also, part of the differences was not very high in absolute fold-change terms. However, we should consider that changes in many biologically relevant lipids, such as specific CEs, are in the 1.5- to 2-fold change.^54^ The possible identification of odd-chain numbered lipids is an intriguing aspect of our study, although it would be important to undertake further mass spectrometric experiments to try to establish if our annotation is indeed correct. These are species with shallow abundance, and the reported changes, though significant, should take into account the low levels and their potential relationship with a low abundancy threshold used here, employed to enhance the chances of finding differential lipids. As usual, all biomarker studies need to be replicated in a more extensive series and confirmed in different setups. We acknowledge also as a limitation the relatively low annotation of many differential lipids. This caveat is due to technical limitations of the available MS. Indeed, not all databases are complete but updated continuously. As we offer the chromatographic and mass spectrometric characteristics of differential lipids, we envisage that independent researchers would further clarify these potential biomarkers' identity. Some of the proposed annotations do not cover complete fatty acyl composition and do not allow us to discuss different potential mechanisms regarding exact fatty acid remodelling and its potential interaction with ALS pathophysiology. Even accounting for this lack of acyl chain elucidation, the significant findings reported in this report are based on the occurrence of specific lipids as candidates for further research in a biomarker for ALS prognosis. Furthermore, several factors validate the current approach: (i) we found common molecules with previously unrelated cohorts of ALS cases, and (ii) the present results converge in previously discovered pathways, such as glycerolipid homeostasis, hypermetabolism, and sphingolipid changes. Overall, our study offers lines for further investigation of lipidome homeostasis in ALS.

## Supplementary material

Supplementary material is available at *Brain Communications* online.

## Supplementary Material

fcab143_Supplementary_Data

## Abbreviations

ALS =: amyotrophic lateral sclerosis
AUC =: area under the curve
BBB =: blood-to-brain barrier
BMI =: body to mass index
CE =: cholesteryl ester
Cer =: ceramide
FDR =: false discovery rate
FP =: fast progressors
LC/MS =: liquid chromatography coupled to mass spectrometry
MTBE =: methyl tert-butyl ether
NfL =: neurofilament light chain
NP =: normal progressors
PA =: phosphatidic acid
PC =: phosphatidylcholine
PCA =: principal component analyses
PE =: Phosphatidylethanolamine
PG =: phosphatidylglycerol
PGE2 =: prostaglandin E2
PI =: phosphatidylinositol
PS =: phosphatidylserine
QC =: quality control
TGs =: triacylglycerides
