# Supplementary material for: Lipidomic traits of plasma and cerebrospinal fluid in amyotrophic lateral sclerosis correlate with disease progression
Source: Brain Commun. 2021 Jun 26;3(3):fcab143. doi: 10.1093/braincomms/fcab143 (PMC8361390; doi:10.1093/braincomms/fcab143)

SUPPLEMENTAL RESULTS

**Supplemental table 1. Expanded characteristics of non-ALS (CTL) samples donors**

| Sex | Ethnicity | Age | Diagnosis |
| --- | --- | --- | --- |
| M | White | 69 | CTL |
| M | White | 44 | PNP |
| M | White | 56 | GBS |
| M | White | 56 | CTL |
| M | White | 80 | CTL |
| F | White | 64 | PNP |
| F | White | 75 | PNP |
| M | White | 51 | CTL |
| F | White | 63 | CTL |
| F | White | 60 | CTL |

M: male, F: female, CTL: self-resolved neurological symptoms; PNP: peripheral polyneuropathy; GBS: Guillain-Barre Syndrome

**Supplemental table 2. Expanded characteristics of ALS patients**

| Sex | Ethnicity | Onset | Age at onset | Days onset-diagnosis | El Elcorial/Arlie at diagnosis | Familiar history | C9orf72 | Other genes |
| --- | --- | --- | --- | --- | --- | --- | --- | --- |
| M | White | Spinal | 51 | 272 | Definite ALS | No | 2&5 | SOD1 normal |
| F | White | Bulbar | 77 | 258 | Possible ALS/ PBP | No | 2&2 | Not done |
| F | White | Spinal | 65 | 312 | Definite ALS | No | 2&8 | Not done |
| F | White | Spinal | 70 | 69 | Definite ALS | No | 2&8 | Not done |
| F | White | Bulbar | 67 | 211 | Definite ALS | No | Not done | Not done |
| M | White | Bulbar | 49 | 595 | Definite ALS | No | 2&2 | Not done |
| M | White | Respiratory | 65 | 336 | Definite ALS | No | 2&2 | Not done |
| M | White | Spinal | 67 | 909 | Suspected ALS/PMA | No | 2&7 | Kennedy normal |
| M | White | Spinal | 60 | 154 | Definite ALS | No | 2&8 | Not done |
| F | White | Spinal | 63 | 376 | Suspected ALS/PMA | No | 7&18 | Not done |
| F | White | Bulbar | 70 | 120 | Probable ALS | No | 2&5 | Not done |
| M | White | Spinal | 40 | 31 | Definite ALS | No | 2&2 | SOD1 normal |
| F | White | Spinal | 57 | 292 | Suspected ALS/PMA | Yes | 2&>30 | Not done |
| M | White | Bulbar | 42 | 82 | Definite ALS | No | Not done | Not done |
| M | White | Respiratory | 68 | 322 | Definite ALS | No | 2&10 | Not done |
| F | White | Respiratory | 76 | 162 | Definite ALS | No | 2&5 | Not done |
| M | White | Spinal | 63 | 263 | Definite ALS | No | 2&8 | Not done |
| F | White | Spinal | 40 | 243 | Definite ALS | No | Not done | Not done |
| F | White | Spinal | 45 | 171 | Definite ALS | No | 5&6 | SOD1 normal |
| M | White | Bulbar | 59 | 191 | Possible ALS/PLS | No | 2&2 | Not done |
| M | White | Spinal | 60 | 139 | Definite ALS | No | 2&5 | Not done |
| M | White | Respiratory | 61 | 113 | Definite ALS | No | 8&8 | Not done |
| M | White | Bulbar | 66 | 132 | Definite ALS | No | 2&2 | Not done |

M: male, F: female, PMA:progressive muscular atrophy, PBP: progressive bulbar palsy, PLS: progressive lateral sclerosis

**Supplemental Table 3.** Lipids used for internal and external standardization

| Compound | Reference |
| --- | --- |
| 1,3(*d*5)-dihexadecanoyl-glycerol | 110537, Avanti Polar Lipids |
| 1,3(*d*5)-dihexadecanoyl-2-octadecanoyl-glycerol | 110543, Avanti Polar Lipids |
| 1-hexadecanoyl(*d*31)-2-(9Z-octadecenoyl)-sn-glycero-3-phosphate | 110920, Avanti Polar Lipids |
| 1-hexadecanoyl(*d*31)-2-(9Z-octadecenoyl)-sn-glycero-3-phosphocholine | 110918, Avanti Polar Lipids |
| 1-hexadecanoyl(*d*31)-2-(9Z-octadecenoyl)-sn-glycero-3-phosphoethanolamine | 110921, Avanti Polar Lipids |
| 1-hexadecanoyl-2-(9Z-octadecenoyl)-sn-glycero-3-phospho-(1'-rac-glycerol-1',1',2',3',3'-*d*5) | 110899, Avanti Polar Lipids |
| 1-hexadecanoyl(*d*31)-2-(9Z-octadecenoyl)-sn-glycero-3-phospho-myo-inositol | 110923, Avanti Polar Lipids |
| 1-hexadecanoyl(*d*31)-2-(9Z-octadecenoyl)-sn-glycero-3-[phospho-L-serine] | 110922, Avanti Polar Lipids |
| 26:0-*d*4 Lyso PC | 860389, Avanti Polar Lipids |
| 18:1 Cholesterol (*d*7) ester | 111015, Avanti Polar Lipids |
| cholest-5-en-3ß-ol(*d*7) | LM-4100, Avanti Polar Lipids |
| D-erythro-sphingosine-*d*7 | 860657, Avanti Polar Lipids |
| D-erythro-sphingosine-*d*7-1-phosphate | 860659, Avanti Polar Lipids |
| N-palmitoyl-*d*31-D-erythro-sphingosine | 868516, Avanti Polar Lipids |
| N-palmitoyl-*d*31-D-erythro-sphingosylphosphorylcholine | 868584, Avanti Polar Lipids |
| Octadecanoic acid-2,2-*d*2 | 19905-58-9, Sigma Aldrich |

Lipid standards consisting of isotopically labelled lipids were used for external standardization (ie, lipid family assignment) and internal standardization (ie, for adjustment of potential inter- and intra-assay variances). Stock solutions were prepared by dissolving lipid standards in methyl tert-butyl ether at a concentration of 1 mg/mL, and working solutions were diluted to 2.5 μg/mL in methyl tert-butyl ether.

**Supplemental Table 4: Lipid species differentially expressed in plasma and CSF in control (CTL) and amyotrophic lateral sclerosis (ALS) cases.**

| Biofluid | Family | Lipid | p-value | FDR corrected p-value | Regulation (ALS vs CTL) | Mass to charge ratio (m/z) | Retention time (min) | Median fold change (ALS vs CTL) | Ionization |
| --- | --- | --- | --- | --- | --- | --- | --- | --- | --- |
| PLA | FA | Dimethyl-PGE2 | 0.035 | 0.639 | up | 379.257 | 4.1 | >10 | - |
|  |  | FA(18:1) | 0.050 | 0.674 | up | 281.2535 | 3.4 | 1.79 | - |
|  |  | FA(18:3) | 0.049 | 0.674 | up | 337.2397 | 3.2 | >10 | - |
|  | GL | DG(34:1) | 0.013 | 0.569 | up | 577.5078 | 6.6 | 1.13 | + |
|  |  | DG(36:4) | 0.026 | 0.609 | up | 634.5283 | 7.5 | >10 | + |
|  |  | DG(38:3) | 0.002 | 0.401 | down | 645.538 | 8.6 | 0.91 | - |
|  |  | DG(42:8) | 0.025 | 0.609 | down | 691.5471 | 8.6 | 0.88 | - |
|  |  | TG(42:0)* | 0.041 | 0.642 | up | 740.6567 | 9.5 | >10 | + |
|  |  | TG(44:0) | 0.011 | 0.569 | up | 768.691 | 9.7 | 2.65 | + |
|  |  | TG(44:1)* | 0.033 | 0.635 | up | 766.6813 | 9.5 | 2.33 | + |
|  |  | TG(48:4) | 0.015 | 0.569 | up | 799.6706 | 9.7 | 2.30 | + |
|  |  | TG(48:8) | 0.020 | 0.569 | down | 849.6337 | 9.7 | <0.1 | - |
|  |  | TG(52:3)* | 0.020 | 0.569 | up | 874.7763 | 10.0 | 1.39 | + |
|  |  | TG(52:4)* | 0.038 | 0.640 | up | 872.7548 | 9.8 | 1.65 | + |
|  |  | TG(52:6) | 0.048 | 0.668 | up | 851.7 | 9.8 | 1.44 | + |
|  |  | TG(54:8) | 0.042 | 0.642 | up | 892.7281 | 9.5 | >10 | + |
|  |  | TG(55:1) | 0.046 | 0.654 | up | 885.8226 | 10.2 | 2.52 | + |
|  |  | TG(56:5) | 0.020 | 0.569 | up | 891.7995 | 10.1 | 1.53 | + |
|  |  | TG(58:11) | 0.044 | 0.642 | up | 907.7407 | 9.4 | >10 | + |
|  |  | TG(58:3) | 0.005 | 0.499 | up | 958.8625 | 10.0 | 1.63 | + |
|  |  | TG(60:7) | 0.019 | 0.569 | down | 959.8138 | 10.0 | <0.1 | - |
|  |  | TG(63:3) | 0.039 | 0.640 | up | 993.9211 | 10.5 | 3.03 | + |
|  | GPL | LysoPS(20:3) | 0.014 | 0.569 | up | 530.2978 | 1.2 | >10 | + |
|  |  | NAPE(52:2) | 0.013 | 0.569 | down | 980.7657 | 10.7 | <0.1 | - |
|  |  | PA(O-42:0) | 0.005 | 0.499 | up | 792.6942 | 6.5 | 2.29 | + |
|  |  | PA(P-36:0) | 0.028 | 0.619 | up | 689.546 | 6.1 | 1.29 | + |
|  |  | PA(P-38:0) | 0.043 | 0.642 | up | 717.5747 | 6.7 | >10 | + |
|  |  | PA(P-39:1) | 0.002 | 0.451 | up | 729.5785 | 6.5 | 1.41 | + |
|  |  | PC(32:1) | 0.041 | 0.642 | up | 732.5371 | 6.6 | 1.94 | + |
|  |  | PC(34:2) | 0.044 | 0.642 | up | 758.5547 | 6.7 | 1.27 | + |
|  |  | PC(36:4)* | 0.011 | 0.569 | up | 782.5592 | 6.7 | 1.46 | + |
|  |  | PC(38:5) | 0.038 | 0.640 | up | 808.5684 | 6.7 | 1.33 | + |
|  |  | PC(38:7) | 0.028 | 0.617 | down | 804.5665 | 7.8 | <0.1 | + |
|  |  | PC(O-28:0) | 0.026 | 0.609 | down | 708.5456 | 8.6 | 0.81 | - |
|  |  | PC(P-38:4) | 0.022 | 0.603 | up | 794.5881 | 7.1 | >10 | + |
|  |  | PC(P-40:7) | 0.034 | 0.635 | up | 816.6064 | 6.7 | 1.12 | + |
|  |  | PE(40:9) | 0.025 | 0.609 | up | 803.5305 | 4.8 | >10 | + |
|  |  | PE(42:1) | 0.023 | 0.609 | up | 852.6391 | 7.1 | >10 | + |
|  |  | PG(34:1)* | <0.001 | 0.299 | up | 766.5472 | 6.6 | 1.1 | + |
|  |  | PG(36:4) | 0.006 | 0.499 | down | 829.5249 | 6.6 | 0.86 | - |
|  |  | PI(38:4) | 0.005 | 0.499 | up | 904.5723 | 6.6 | 4.37 | + |
|  |  | PI(O-34:1) | 0.001 | 0.299 | up | 840.6116 | 6.6 | 1.59 | + |
|  |  | PI(P-36:0) | 0.009 | 0.556 | up | 868.6423 | 7.2 | 1.51 | + |
|  | SP | Cer(d38:3) | 0.034 | 0.635 | down | 612.5188 | 8.1 | <0.1 | + |
|  |  | Cer(d40:1) | 0.026 | 0.609 | up | 622.6058 | 8.5 | >10 | + |
|  |  | Cer(t42:0) | 0.037 | 0.640 | down | 726.6811 | 7.1 | 0.93 | - |
|  |  | LacCer(d32:1) | 0.038 | 0.640 | up | 834.5884 | 7.1 | 1.4 | + |
|  |  | NeuAc2-3Galβ-Cer(d42:1) | 0.020 | 0.569 | down | 1101.783 | 10.1 | <0.1 | - |
|  |  | PI-Cer(d36:0) | 0.043 | 0.642 | up | 810.5883 | 7.2 | 1.7 | + |
|  |  | SM(d34:2) | 0.031 | 0.635 | up | 701.5473 | 5.9 | 1.24 | + |
| CSF | GL | DG(44:3) | 0.019 | 0.569 | down | 789.6497 | 9.8 | <0.1 | - |
|  |  | MG(18:0)a | <0.001 | 0.073 | down | 417.3287 | 4.8 | <0.1 | - |
|  |  | TG(64:8) | 0.037 | 0.640 | down | 1073.867 | 10.7 | <0.1 | - |

Annotation is based on exact mass, retention time and isotopic distribution *: confirmed by MS/MS, ^a^: FDR corrected p-value < 0.05.

FA: fatty Acyls, GL: glycerolipids, GPL: glycerophospholipids, SP: sphingolipids, SL: sterol lipids, PGE2: prostaglandin E2, MG: monoacylglycerol, DG: diacylglycerol, TG: triglyceride, NAPE: N-Acetyl.phosphatidylethanolamine, PA: Phosphatidic acid, PC: Phosphatidylcholine, PE: Phosphatidylethanolamine, PG: Phosphatidylglycerol, PI: Phosphatidylinositol, PS: Phosphatidylserine , Cer: Ceramide, NeuAc: N-Acetyl-Neuraminic Acid, CE: Cholesteryl ester. Ether lipids may be found as ‘plasmanyl’ (also termed alkyl ethers, and represented by the ‘O-‘ prefix), and as ‘plasmenyl’ (also termed alkenyl ethers or plasmalogens, and represented by the ‘P-‘ prefix). Lyso prefix refers to lysophospholipids.

Supplemental Table 5: Plasma and CSF lipid species which correlate with disease onset

| Biofluid | Family | Lipid | p-value | FDR corrected P value | Post-hoc | Mass to charge ratio (m/z) | Retention time (min) | Median Fold Change (Resp vs Bulb) | Median Fold Change (Resp vs Spin) | Median Fold Change (Bulb vs Spin) | Ionization |
| --- | --- | --- | --- | --- | --- | --- | --- | --- | --- | --- | --- |
| PLA | GL | DG(34:1) | 0.050 | 1 | BULBAR - SPINAL, RESPIRATORY - SPINAL | 577.5078 | 6.6 | - | 1.26 | 1.20 | + |
|  |  | DG(35:3) | 0.024 | 1 | BULBAR - RESPIRATORY, RESPIRATORY - SPINAL | 605.5144 | 7.6 | 1.46 | 1.41 | - | + |
|  |  | DG(38:1) | 0.003 | 1 | BULBAR - SPINAL, RESPIRATORY - SPINAL | 668.6085 | 7.3 | - | 1.59 | 1.27 | + |
|  |  | TG(58:1) | 0.020 | 1 | BULBAR - SPINAL, RESPIRATORY - SPINAL | 927.8917 | 10.2 | - | <0.1 | <0.1 | + |
|  |  | TG(58:11) | 0.049 | 1 | BULBAR - RESPIRATORY | 907.7407 | 9.4 | <0.1 | - | - | + |
|  |  | TG(58:7) | 0.045 | 1 | RESPIRATORY - SPINAL | 915.8021 | 10.0 | - | 1.34 | - | + |
|  |  | TG(60:8) | 0.041 | 1 | BULBAR - RESPIRATORY, BULBAR - SPINAL | 959.778 | 9.5 | 0.53 | - | 2.68 | + |
|  | GPL | LysoPC(22:1) | 0.021 | 1 | BULBAR - SPINAL, RESPIRATORY - SPINAL | 578.4207 | 0.9 | - | 1.27 | 1.14 | + |
|  |  | PA(O-42:0) | 0.018 | 1 | BULBAR - RESPIRATORY, RESPIRATORY - SPINAL | 792.6942 | 6.5 | 1.41 | 1.75 | - | + |
|  |  | PA(P-42:6) | 0.047 | 1 | BULBAR - RESPIRATORY, RESPIRATORY - SPINAL | 759.5266 | 8.6 | 0.92 | 0.94 | - | - |
|  |  | PC(48:1) | 0.046 | 1 | BULBAR - SPINAL, RESPIRATORY - SPINAL | 978.8031 | 9.6 | - | <0.1 | <0,1 | + |
|  |  | PC(P-38:4) | 0.047 | 1 | BULBAR - RESPIRATORY, BULBAR - SPINAL | 794.5881 | 7.1 | 0.39 | - | 1.24 | + |
|  |  | PS(40:4) | 0.036 | 1 | BULBAR - SPINAL, RESPIRATORY - SPINAL | 898.5864 | 6.7 | - | 0.78 | 0.81 | - |
|  | SP | Cer(d34:1) | 0.018 | 1 | BULBAR - SPINAL, RESPIRATORY - SPINAL | 555.5384 | 9.2 | - | 1.35 | 1.19 | + |
| CSF | GL | TG(54:4)* | 0.027 | 1 | BULBAR - RESPIRATORY, RESPIRATORY - SPINAL | 900.7938 | 10.2 | 0.32 | 0.35 | - | + |
|  |  | TG(56:9) | 0.029 | 1 | RESPIRATORY - SPINAL | 901.7339 | 9.9 | - | <0.1 | - | + |
|  | GPL | PA(33:3) | 0.027 | 1 | BULBAR - RESPIRATORY, RESPIRATORY - SPINAL | 655.4273 | 5.2 | 1.30 | >10 | - | - |
|  | SP | Ganglioside GA2 (d40:1) | 0.036 | 1 | BULBAR - SPINAL, RESPIRATORY - SPINAL | 1131.773 | 8.0 | - | 0.47 | 0.79 | + |

Annotation is based on exact mass, retention time and isotopic distribution *: confirmed by MS/MS, ^a^: FDR corrected p-value < 0.05. Post-hoc pairs indicate significantly differed pairs. FA: fatty Acyls, GL: glycerolipids, GPL: glycerophospholipids, SP: sphingolipids, MG: monoacylglycerol, DG: diacylglycerol, TG: triglyceride, PA: Phosphatidic acid, PC: Phosphatidylcholine, PE: Phosphatidylethanolamine, PG: Phosphatidylglycerol, PI: Phosphatidylinositol, PS: Phosphatidylserine , Cer: Ceramide, CE: Cholesteryl ester. Ether lipids may be found as ‘plasmanyl’ (also termed alkyl ethers, and represented by the ‘O-‘ prefix), and as ‘plasmenyl’ (also termed alkenyl ethers or plasmalogens, and represented by the ‘P-‘ prefix). Lyso prefix refers to lysophospholipids.

Supplemental table 6. Differences in clinical characteristics according site of disease onset

| Type (n) | Bulbar (6) | Respiratory (4) | Spinal(12) | ANOVA p value |
| --- | --- | --- | --- | --- |
| BMI at diagnosis(Kg/m^2^) | 26.28±2.53 | 25.96±4.54 | 23.18±3.20 | 0.13 |
| Usual BMI (Kg/m^2^) | 27.17±2.82 | 29.23±4.94 | 20.02±3.45 | 0.035 |
| Minimum BMI (Kg/m^2^) | 24.53±2.28 | 24.29±3.92 | 21.56±4.37 | 0.23 |
| Maximum BMI (Kg/m^2^) | 27.16±2 | 27.08±3.16 | 23.63±3.97 | 0.072 |
| CK (μkat/L) | 4.31±3.65 | 1.75±0.625 | 6.70±3.72 | 0.049 |

Supplemental Table 7. Plasma and CSF lipid species which correlate with disease progression.

| Biofluid | Family | Lipid | p-value | FDR corrected p-value | Regulation (FP vs NP) | Mass to charge ratio (m/z) | Retention time (min) | Median fold change (FP vs NP) | Ionization |
| --- | --- | --- | --- | --- | --- | --- | --- | --- | --- |
| PLA | FA | FA(18:0) | 0.049 | 0.365 | up | 283.2717 | 4.1 | 2.20 | - |
|  | GL | DG(36:2)* | 0.035 | 0.339 | down | 638.5609 | 8.1 | 0.55 | + |
|  |  | DG(36:3) | 0.043 | 0.351 | down | 617.4987 | 8.1 | 0.26 | + |
|  |  | DG(37:4) | 0.046 | 0.358 | down | 629.5039 | 8.1 | 0.68 | - |
|  |  | DG(38:5) | 0.024 | 0.316 | down | 643.5122 | 8.2 | 0.56 | + |
|  |  | DG(44:9) | 0.024 | 0.316 | down | 701.5707 | 7.8 | <0.1 | + |
|  |  | DG(46:0) | 0.043 | 0.351 | down | 765.7308 | 8.8 | 0.53 | + |
|  |  | DG(46:1) | 0.005 | 0.230 | down | 763.7114 | 8.5 | 0.62 | + |
|  |  | TG(42:2) | 0.042 | 0.351 | down | 736.6293 | 8.1 | 0.64 | + |
|  |  | TG(44:0) | 0.020 | 0.309 | down | 768.691 | 9.7 | 0.51 | + |
|  |  | TG(44:1)* | 0.010 | 0.263 | down | 766.6813 | 9.5 | 0.41 | + |
|  |  | TG(46:1) | 0.010 | 0.263 | down | 794.7108 | 9.7 | 0.43 | + |
|  |  | TG(46:2)* | 0.006 | 0.242 | down | 792.6965 | 9.5 | 0.43 | + |
|  |  | TG(48:1)* | 0.042 | 0.351 | down | 822.7443 | 9.9 | 0.44 | + |
|  |  | TG(48:2) | 0.024 | 0.316 | down | 820.7229 | 9.7 | 0.41 | + |
|  |  | TG(48:3) | 0.031 | 0.321 | down | 818.7063 | 9.6 | 0.36 | + |
|  |  | TG(48:4) | 0.022 | 0.316 | down | 799.6706 | 9.7 | 0.55 | + |
|  |  | TG(48:5) | 0.039 | 0.351 | down | 797.6547 | 9.5 | 0.48 | + |
|  |  | TG(50:3)* | 0.042 | 0.351 | down | 846.7442 | 9.8 | 0.47 | + |
|  |  | TG(50:4) | 0.016 | 0.285 | down | 844.7229 | 9.6 | 0.52 | + |
|  |  | TG(50:5) | 0.013 | 0.273 | down | 807.7021 | 9.5 | <0.1 | + |
|  |  | TG(52:2) | 0.048 | 0.361 | down | 876.7893 | 10.1 | 0.63 | + |
|  |  | TG(52:5) | 0.029 | 0.320 | down | 870.7442 | 9.7 | 0.61 | + |
|  |  | TG(52:6)* | 0.044 | 0.351 | down | 868.7279 | 9.5 | 0.49 | + |
|  |  | TG(52:8) | 0.011 | 0.267 | down | 845.6733 | 8.0 | 0.60 | - |
|  |  | TG(53:8) | 0.012 | 0.272 | down | 859.6985 | 8.2 | 0.69 | - |
|  |  | TG(54:2) | 0.015 | 0.285 | down | 904.8138 | 9.7 | <0.1 | + |
|  |  | TG(54:5) | 0.029 | 0.320 | down | 898.7699 | 9.8 | 0.70 | + |
|  |  | TG(54:7) | 0.046 | 0.358 | down | 859.7341 | 9.6 | 0.42 | + |
|  |  | TG(55:4) | 0.012 | 0.272 | up | 955.8057 | 10.2 | >10 | - |
|  |  | TG(56:12) | 0.015 | 0.285 | down | 953.7029 | 8.0 | <0.1 | - |
|  |  | TG(56:2) | 0.012 | 0.272 | down | 932.853 | 10.4 | 0.34 | + |
|  |  | TG(56:3) | 0.031 | 0.320 | down | 930.8358 | 9.8 | 0.49 | + |
|  |  | TG(56:5)* | 0.042 | 0.351 | down | 926.8061 | 10.1 | 0.81 | + |
|  |  | TG(56:8) | 0.020 | 0.309 | down | 885.7473 | 9.6 | 0.73 | + |
|  |  | TG(58:1) | 0.029 | 0.320 | up | 927.8917 | 10.2 | >10 | + |
|  |  | TG(60:3) | 0.020 | 0.309 | down | 986.8921 | 10.1 | 0.77 | + |
|  |  | TG(60:4) | 0.042 | 0.351 | down | 984.8815 | 10.0 | 0.77 | + |
|  |  | TG(60:5) | 0.023 | 0.316 | down | 982.8655 | 9.8 | 0.74 | + |
|  |  | TG(60:6) | 0.026 | 0.320 | down | 980.8497 | 9.7 | <0.1 | + |
|  |  | TG(63:3) | 0.002 | 0.204 | down | 993.9211 | 10.5 | 0.72 | + |
|  |  | TG(65:12) | 0.024 | 0.316 | up | 1019.803 | 10.0 | 1.41 | - |
|  |  | TG(65:4) | 0.005 | 0.230 | down | 1019.9396 | 10.5 | 0.70 | + |
|  |  | TG(65:5) | 0.006 | 0.242 | down | 1017.9198 | 10.3 | 0.80 | + |
|  | GPL | LysoPC(16:0)* | 0.029 | 0.320 | down | 496.328 | 2.5 | 0.58 | + |
|  |  | LysoPC(18:0)* | 0.004 | 0.214 | down | 524.3606 | 3.3 | 0.49 | + |
|  |  | LysoPC(18:1) | 0.015 | 0.285 | down | 522.3427 | 2.6 | 0.61 | + |
|  |  | LysoPC(18:2)* | 0.037 | 0.348 | down | 520.3294 | 2.1 | 0.63 | + |
|  |  | PA(P-42:6) | 0.005 | 0.230 | down | 759.5266 | 8.6 | 0.95 | - |
|  |  | PC(32:1) | 0.035 | 0.339 | down | 732.5371 | 6.6 | 0.63 | + |
|  |  | PC(32:2) | 0.018 | 0.309 | down | 730.5236 | 6.1 | 0.61 | + |
|  |  | PC(34:1) | 0.008 | 0.248 | down | 760.5694 | 7.1 | 0.58 | + |
|  |  | PC(34:2) | 0.003 | 0.204 | down | 758.5547 | 6.7 | 0.68 | + |
|  |  | PC(36:1) | 0.016 | 0.290 | down | 788.6042 | 7.6 | <0.1 | + |
|  |  | PC(36:2) | 0.001 | 0.113 | down | 786.5912 | 7.2 | 0.58 | + |
|  |  | PC(36:4)*^a^ | <0.001 | 0.070 | down | 782.5592 | 6.6 | 0.52 | + |
|  |  | PC(38:5) | <0.001 | 0.102 | down | 808.5684 | 6.7 | 0.59 | + |
|  |  | PC(42:6) | 0.001 | 0.113 | down | 844.641 | 7.2 | 0.50 | + |
|  |  | PC(O-28:0) | 0.034 | 0.339 | up | 708.5456 | 8.6 | >10 | - |
|  |  | PC(P-38:3) | 0.040 | 0.351 | down | 794.5959 | 8.7 | <0.1 | - |
|  |  | PC(P-38:4) | 0.017 | 0.293 | down | 794.5881 | 7.1 | 0.74 | + |
|  |  | PC(P-40:6) | 0.012 | 0.272 | down | 818.6213 | 7.1 | 0.50 | + |
|  |  | PC(P-40:7) | 0.005 | 0.230 | down | 816.6064 | 6.7 | 0.80 | + |
|  |  | PC(P-42:6) | <0.001 | 0.102 | down | 846.6499 | 7.6 | <0.1 | + |
|  |  | PE(12:0) | 0.022 | 0.316 | up | 429.2343 | 4.8 | >10 | + |
|  |  | PE(36:2) | 0.048 | 0.361 | down | 742.5402 | 7.3 | <0.1 | - |
|  |  | PE(38:1) | 0.041 | 0.351 | down | 774.5842 | 7.4 | <0.1 | + |
|  |  | PE(38:2) | 0.024 | 0.316 | down | 772.5729 | 7.0 | 0.73 | + |
|  |  | PE(40:5) | 0.008 | 0.248 | down | 811.5937 | 7.2 | 0.20 | + |
|  |  | PE(40:9) | 0.029 | 0.320 | down | 803.5305 | 4.8 | 0.77 | + |
|  |  | PE(42:1) | 0.002 | 0.204 | down | 852.6391 | 7.0 | <0.1 | + |
|  |  | PE(O-42:4) | <0.001 | 0.102 | down | 808.621 | 8.8 | 0.59 | - |
|  |  | PE(P-36:4) | 0.046 | 0.358 | down | 722.5184 | 7.0 | 0.69 | - |
|  |  | PE(P-38:4) | 0.015 | 0.285 | down | 752.545 | 7.5 | 0.68 | + |
|  |  | PE(P-38:4)* | 0.014 | 0.278 | down | 750.5468 | 7.5 | 0.62 | - |
|  |  | PG(34:1)* | 0.035 | 0.339 | down | 766.5472 | 6.6 | 0.85 | + |
|  |  | PG(36:4) | 0.010 | 0.263 | up | 829.5249 | 6.6 | 1.13 | - |
|  |  | PG(P-34:0) | 0.003 | 0.204 | down | 733.5525 | 5.8 | 0.60 | - |
|  |  | PI(O-34:1) | 0.004 | 0.214 | down | 840.6116 | 6.6 | 0.63 | + |
|  |  | PI(O-36:0) | 0.008 | 0.248 | down | 870.6603 | 7.4 | 0.14 | + |
|  |  | PI(O-36:2) | 0.044 | 0.351 | down | 866.6206 | 6.7 | 0.34 | + |
|  |  | PI(P-36:0)^a^ | <0.001 | 0.047 | down | 868.6423 | 7.2 | 0.58 | + |
|  |  | PS(40:1) | 0.042 | 0.351 | down | 844.6154 | 7.3 | 0.87 | - |
|  |  | PS(40:2) | 0.023 | 0.316 | up | 844.6046 | 7.8 | >10 | + |
|  | SP | Cer(d42:1) | 0.001 | 0.102 | down | 650.6322 | 8.8 | 0.56 | + |
|  |  | Cer(t42:0) | 0.029 | 0.320 | up | 726.6811 | 7.1 | 1.10 | - |
|  |  | Ganglioside GM3(d40:2) | 0.005 | 0.230 | up | 1235.8322 | 7.2 | 1.75 | + |
|  |  | LacCer(d34:1) | 0.045 | 0.357 | up | 862.61 | 6.3 | >10 | + |
|  |  | PI-Cer(d36:0) | 0.020 | 0.309 | down | 810.5883 | 7.2 | 0.58 | + |
|  |  | SM(d32:0) | 0.006 | 0.242 | down | 677.5698 | 7.8 | 0.67 | + |
|  |  | SM(d32:1) | 0.002 | 0.196 | down | 675.5274 | 5.8 | 0.63 | + |
|  |  | SM(d34:1) | 0.013 | 0.272 | down | 703.5589 | 6.4 | 0.80 | + |
|  |  | SM(d34:2) | 0.001 | 0.102 | down | 701.5473 | 5.9 | 0.75 | + |
|  |  | SM(d36:2) | 0.048 | 0.361 | down | 711.5672 | 9.0 | 0.82 | + |
|  |  | SM(d40:1) | 0.036 | 0.346 | down | 787.6505 | 8.0 | 0.67 | + |
|  |  | SM(d42:1) | 0.006 | 0.242 | down | 815.6861 | 8.4 | 0.69 | + |
|  |  | SM(d42:2) | 0.020 | 0.309 | down | 813.6713 | 8.0 | 0.84 | + |
|  | SL | CE(14:1) | 0.006 | 0.242 | down | 653.5382 | 8.1 | 0.63 | - |
|  |  | CE(18:1)* | 0.001 | 0.102 | down | 668.6234 | 10.5 | 0.69 | + |
|  |  | CE(18:2)* | 0.020 | 0.309 | down | 666.6068 | 10.3 | 0.80 | + |
|  |  | CE(20:5) | 0.029 | 0.320 | down | 653.5734 | 8.1 | 0.53 | + |
|  |  | CE(22:5) | 0.027 | 0.320 | down | 697.6127 | 8.7 | 0.64 | - |
| CSF | GL | DG(36:0) | 0.030 | 0.320 | up | 683.5875 | 9.1 | 2.76 | - |
|  |  | DG(42:4) | 0.024 | 0.316 | down | 718.6201 | 5.6 | <0.1 | + |
|  |  | TG(49:1)* | 0.048 | 0.361 | down | 836.7594 | 10.2 | 0.94 | + |
|  |  | TG(50:1)* | 0.049 | 0.361 | down | 850.7746 | 10.3 | 0.73 | + |
|  |  | TG(54:3)* | 0.047 | 0.361 | down | 902.8085 | 10.3 | 0.81 | + |
|  |  | TG(54:4) | 0.037 | 0.348 | down | 941.7935 | 10.3 | <0.1 | - |
|  |  | TG(54:4)* | 0.026 | 0.320 | down | 900.7938 | 10.2 | 0.89 | + |
|  | GPL | PA(33:3) | 0.018 | 0.309 | up | 655.4273 | 5.2 | >10 | - |
|  |  | PS(36:3) | 0.013 | 0.272 | up | 803.5355 | 6.3 | >10 | + |
|  | SP | CerP(34:0) | 0.036 | 0.346 | down | 619.5179 | 8.7 | <0.1 | + |
|  |  | Ganglioside GM3(d36:2) | 0.023 | 0.316 | down | 1179.76 | 6.5 | 0.81 | + |

Annotation is based on exact mass, retention time and isotopic distribution *: confirmed by MS/MS, ^a^: FDR corrected p-value < 0.05.

FP: fast progressors; NP: normal progressors. FA: fatty Acyls, GL: glycerolipids, GPL: glycerophospholipids, SP: sphingolipids, SL: sterol lipids, DG: diacylglycerol, TG: triglyceride, PA: Phosphatidic acid, PC: Phosphatidylcholine, PE: Phosphatidylethanolamine, PG: Phosphatidylglycerol, PI: Phosphatidylinositol, PS: Phosphatidylserine , Cer: Ceramide, CE: Cholesteryl ester. Ether lipids may be found as ‘plasmanyl’ (also termed alkyl ethers, and represented by the ‘O-‘ prefix), and as ‘plasmenyl’ (also termed alkenyl ethers or plasmalogens, and represented by the ‘P-‘ prefix). Lyso prefix refers to lysophospholipids.

Supplemental table 8. Differences in clinical characteristics according progression rate

| Progression rate (n) | Fast progression (15) | Normal progression (8) | p value for t-test |
| --- | --- | --- | --- |
| BMI at diagnosis(Kg/m2) | 25.04±3.81 | 24.25±3.46 | 0.61 |
| Usual BMI (Kg/m2) | 25.81±4.56 | 26.19±3.72 | 0.83 |
| Minimum BMI (Kg/m2) | 21.77±3.75 | 24.44±3.88 | 0.11 |
| Maximum BMI (Kg/m2) | 24.37±3.68 | 26.51±3.58 | 0.17 |
| CK (μkat/L) | 5.54±3.90 | 5.10±3.86 | 0.81 |

**Supplemental Figure 1** Lipidomic profiles in plasma (A) and CSF (B) in ALS patients in comparison with non-ALS individuals (CTL). Left panels: lipids from each fraction define a graph of principal component analysis (PCA). Right panels: Relevant molecular pathways, inferred by mapping differentially downregulated (C) or upregulated (D) lipids in plasma. The size of the nodes (pathways) is proportional to the total number of metabolites belonging to the pathway; the color intensity is inversely proportional to the p-value of the enrichment analyses; the edge width, directly proportional to the number of common metabolites, and the edge color intensity, directly proportional to the number of lipids differentially regulated in ALS vs CTL cases. These results represent lipids detected by negative ionization.

**
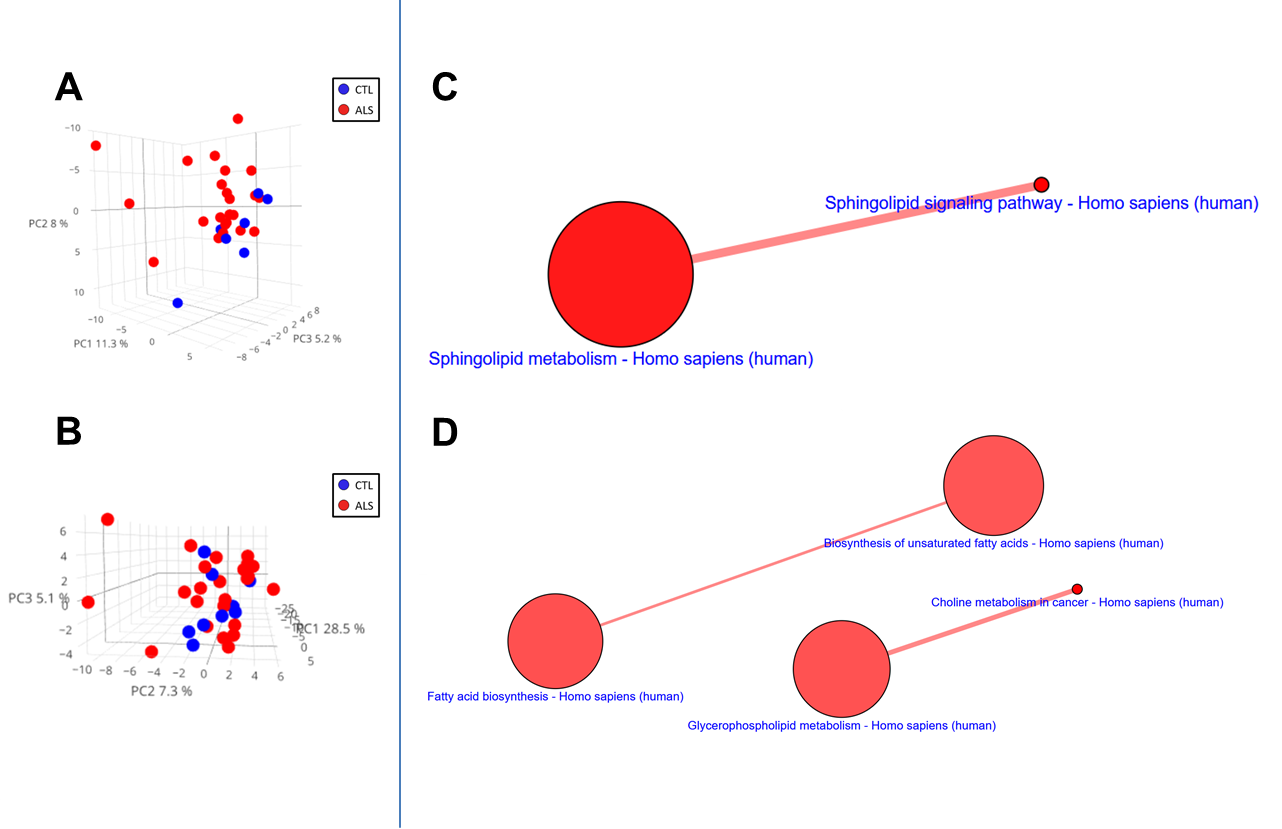
**

**Supplemental Figure 2** Plasma (A and C) and CSF (B and D) lipidomic profile of ALS patients according to the region of onset (bulbar v.s. spinal v.s. respiratory). Upper panel: Principal component analysis. Lower panel: Hierarchical clusterings using the 25 molecular features with the lowest p-value obtained from univariate statistics. Each segment of the heatmap represents a lipid species colored by its abundance intensity, normalized to an internal standard, log-transformed, and row-normalized using Z-score. The scale from blue to red represents this normalized abundance in MS counts. Lipids are organized in rows. We employed Ward clustering method and Manhattan distance for hierarchical clustering. Samples are organized in columns and ordered according to hierarchical clustering. Dendrograms and sample grouping are displayed by different colors: bulbar in blue, spinal in green, and respiratory in red. Results represent molecular findings detected by positive ionization.

**
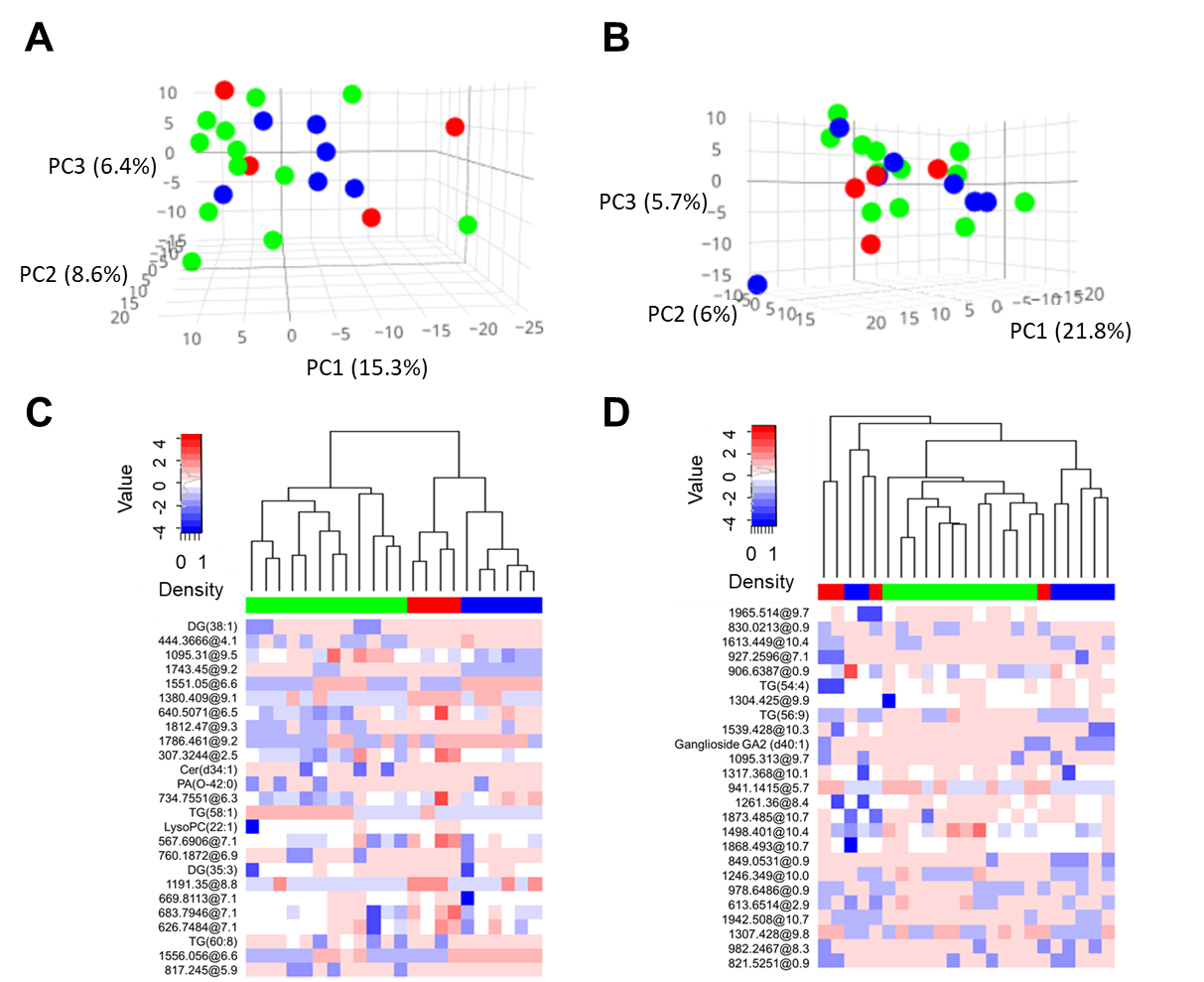
**

**Supplemental Figure 3**. Plasma (A) and CSF (B) lipidomic profile of ALS patients according to the onset (Bulbar vs Spinal vs Respiratory). Principal component analysis. X axis: Principal component 1. Y axis: Principal component 2. Z axis: Principal component 3. Individuals from Bulbar group are represented with blue spheres, individuals from Spinal group with green spheres and individuals from Respiratory group with red spheres. This results represent molecular features detected only in negative ionization.

**
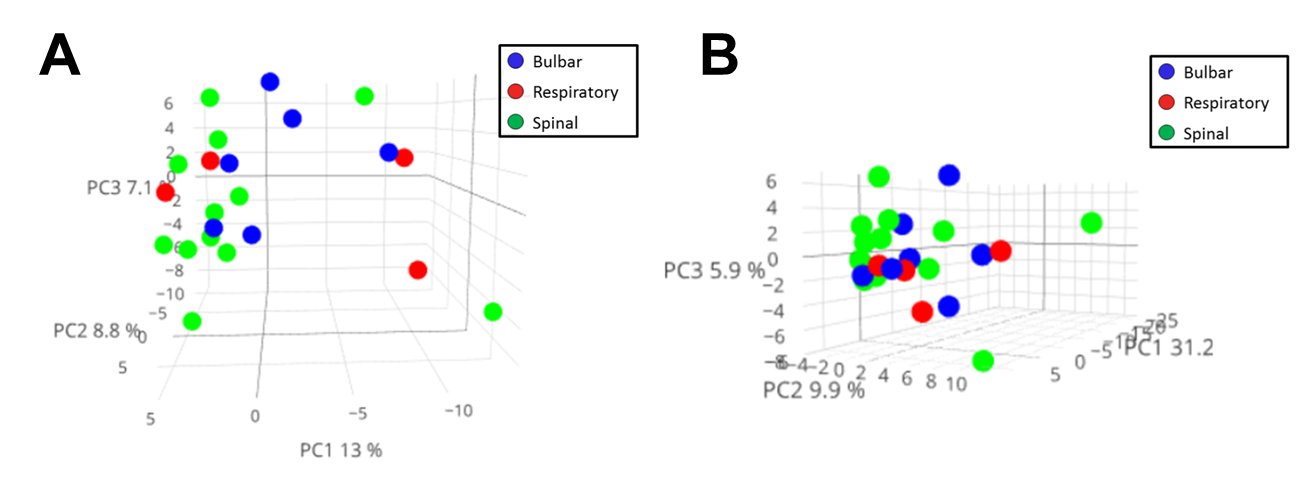
**

**Supplemental Figure 4** Lipidomic profiles in reference to disease progression in ALS. Plasma (A) and CSF (B) lipidomic profiles in fast progressor (FP) v.s. normal progressors (NP). Left panels: Lipids from each fraction define a graph of principal component analysis (PCA) with marked differences in plasma and CSF. Right panels: Relevant molecular pathways, inferred by mapping differentially downregulated (C) or upregulated (D) lipids in plasma. The size of the nodes (pathways) is proportional to the total number of metabolites belonging to the pathway; the color intensity is inversely proportional to the p-value of the enrichment analyses; the edge width is directly proportional to the number of common metabolites, and the edge color intensity, directly proportional to the number of lipids differentially regulated in FP v.s. NP. These results represent lipids detected by negative ionization.

**
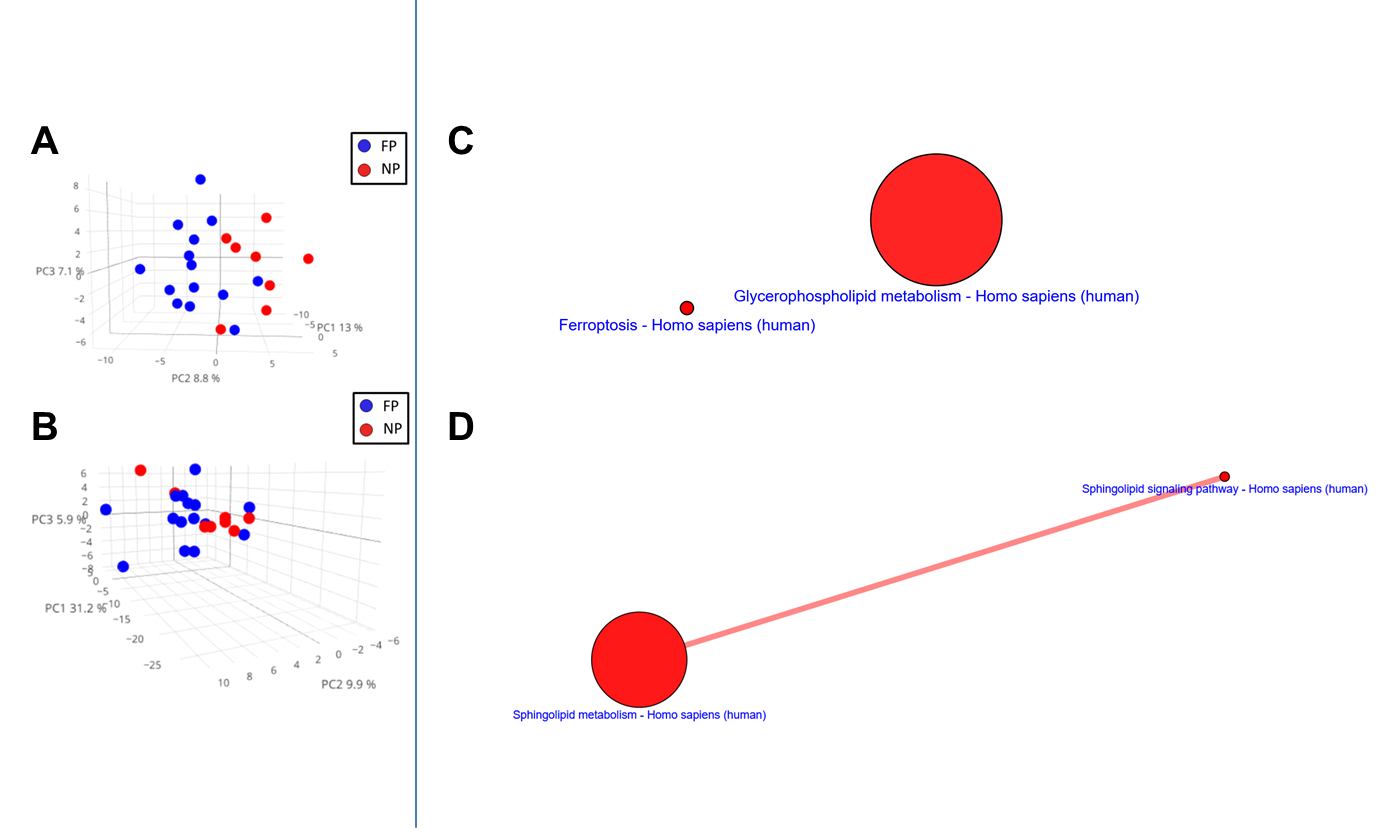
**

**Supplemental Figure 5** Specificity of lipidomic changes between ALS v.s. non-ALS, ALS onset, and ALS progression rate. A) common lipids which are differential in the ALS v.s. non-ALS and in the region of ALS onset. B) common lipids that are differential in the region of ALS onset in FP v.s. NP cases. In both heatmaps, each segment of the graph represents the mean value of the lipid species abundance colored by its abundance intensity, normalized to an internal standard, log-transformed, and row-normalized Z-scores. The scale from blue to red represents these normalized abundances in MS counts. Disease groups are organized in columns.

**
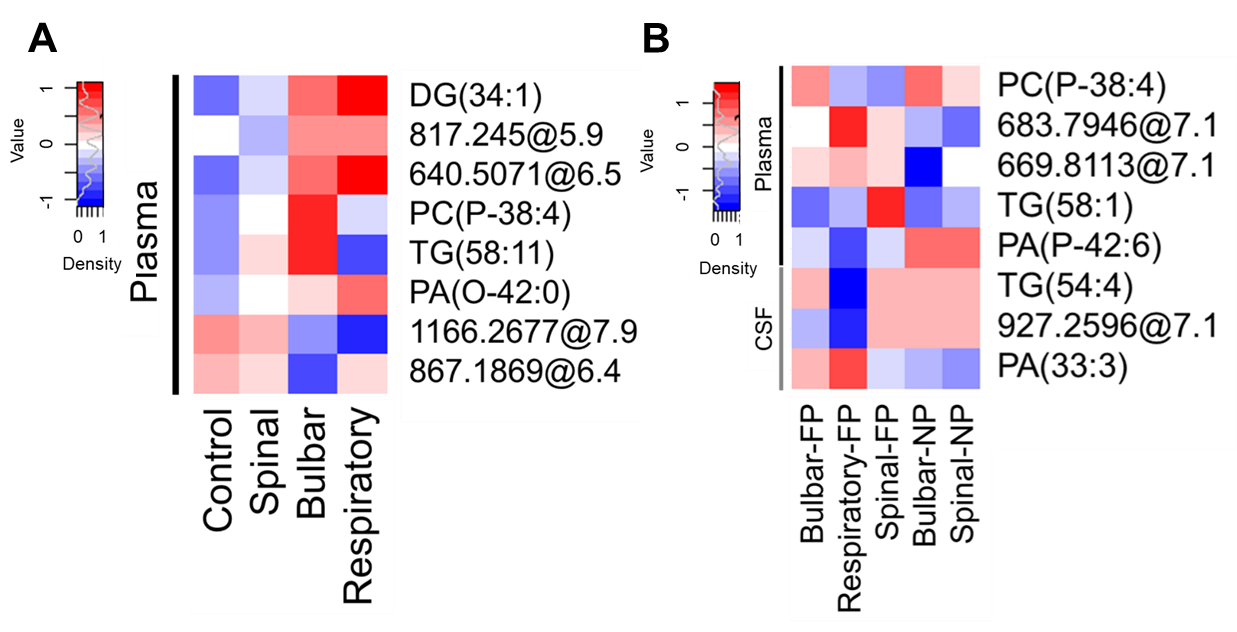
**

**Supplemental Figure 6.** A and B) Receiver Operating Characteristic curve of PC(44:8) and PC(36:4) (species with maximum capacity for discrimination, see main text) for the classification between NP and FP. Levels shown are in MS counts


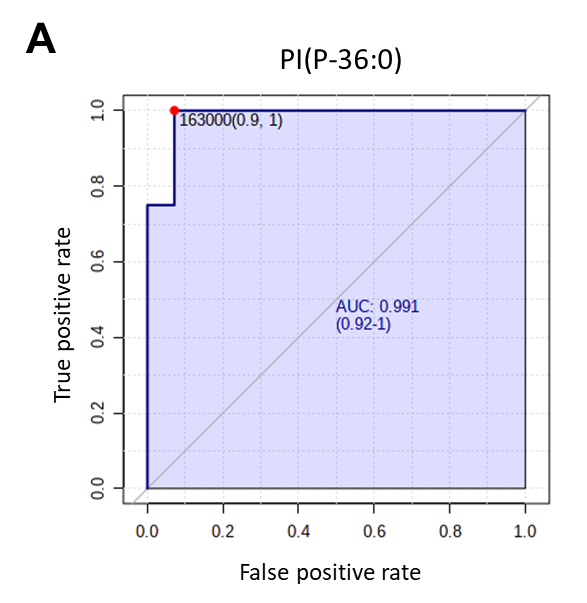

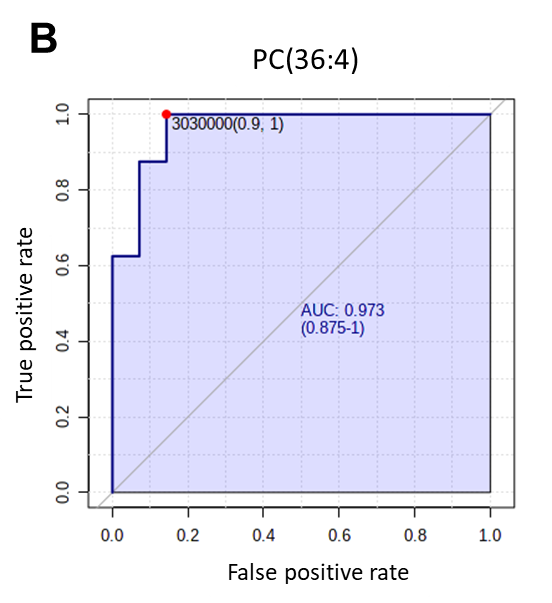

Supplement: fcab143_Supplementary_Data [file fcab143_supplementary_data.zip › Supplementary_material.docx]
